# Association of germline genetic variants with breast cancer-specific survival in patient subgroups defined by clinic-pathological variables related to tumor biology and type of systemic treatment

**DOI:** 10.1186/s13058-021-01450-7

**Published:** 2021-08-18

**Authors:** Anna Morra, Maria Escala-Garcia, Jonathan Beesley, Renske Keeman, Sander Canisius, Thomas U. Ahearn, Irene L. Andrulis, Hoda Anton-Culver, Volker Arndt, Paul L. Auer, Annelie Augustinsson, Laura E. Beane Freeman, Heiko Becher, Matthias W. Beckmann, Sabine Behrens, Stig E. Bojesen, Manjeet K. Bolla, Hermann Brenner, Thomas Brüning, Saundra S. Buys, Bette Caan, Daniele Campa, Federico Canzian, Jose E. Castelao, Jenny Chang-Claude, Stephen J. Chanock, Ting-Yuan David Cheng, Christine L. Clarke, NBCS Collaborators, Anne-Lise Børresen-Dale, Kristine K. Sahlberg, Lars Ottestad, Rolf Kåresen, Ellen Schlichting, Marit Muri Holmen, Toril Sauer, Vilde Haakensen, Olav Engebråten, Bjørn Naume, Alexander Fosså, Cecile E. Kiserud, Kristin V. Reinertsen, Åslaug Helland, Margit Riis, Jürgen Geisler, Grethe I. Grenaker Alnæs, Sarah V. Colonna, Fergus J. Couch, Angela Cox, Simon S. Cross, Kamila Czene, Mary B. Daly, Joe Dennis, Thilo Dörk, Laure Dossus, Alison M. Dunning, Miriam Dwek, Diana M. Eccles, Arif B. Ekici, A. Heather Eliassen, Mikael Eriksson, D. Gareth Evans, Peter A. Fasching, Henrik Flyger, Lin Fritschi, Manuela Gago-Dominguez, José A. García-Sáenz, Graham G. Giles, Mervi Grip, Pascal Guénel, Melanie Gündert, Eric Hahnen, Christopher A. Haiman, Niclas Håkansson, Per Hall, Ute Hamann, Steven N. Hart, Jaana M. Hartikainen, Arndt Hartmann, Wei He, Maartje J. Hooning, Reiner Hoppe, John L. Hopper, Anthony Howell, David J. Hunter, ABCTB Investigators, Christine Clarke, Deborah Marsh, Rodney Scott, Robert Baxter, Desmond Yip, Jane Carpenter, Alison Davis, Nirmala Pathmanathan, Peter Simpson, J. Dinny Graham, Mythily Sachchithananthan, kConFab Investigators, Agnes Jager, Anna Jakubowska, Wolfgang Janni, Esther M. John, Audrey Y. Jung, Rudolf Kaaks, Machteld Keupers, Cari M. Kitahara, Stella Koutros, Peter Kraft, Vessela N. Kristensen, Allison W. Kurian, James V. Lacey, Diether Lambrechts, Loic Le Marchand, Annika Lindblom, Martha Linet, Robert N. Luben, Jan Lubiński, Michael Lush, Arto Mannermaa, Mehdi Manoochehri, Sara Margolin, John W. M. Martens, Maria Elena Martinez, Dimitrios Mavroudis, Kyriaki Michailidou, Roger L. Milne, Anna Marie Mulligan, Taru A. Muranen, Heli Nevanlinna, William G. Newman, Sune F. Nielsen, Børge G. Nordestgaard, Andrew F. Olshan, Håkan Olsson, Nick Orr, Tjoung-Won Park-Simon, Alpa V. Patel, Bernard Peissel, Paolo Peterlongo, Dijana Plaseska-Karanfilska, Karolina Prajzendanc, Ross Prentice, Nadege Presneau, Brigitte Rack, Gad Rennert, Hedy S. Rennert, Valerie Rhenius, Atocha Romero, Rebecca Roylance, Matthias Ruebner, Emmanouil Saloustros, Elinor J. Sawyer, Rita K. Schmutzler, Andreas Schneeweiss, Christopher Scott, Mitul Shah, Snezhana Smichkoska, Melissa C. Southey, Jennifer Stone, Harald Surowy, Anthony J. Swerdlow, Rulla M. Tamimi, William J. Tapper, Lauren R. Teras, Mary Beth Terry, Rob A. E. M. Tollenaar, Ian Tomlinson, Melissa A. Troester, Thérèse Truong, Celine M. Vachon, Qin Wang, Amber N. Hurson, Robert Winqvist, Alicja Wolk, Argyrios Ziogas, Hiltrud Brauch, Montserrat García-Closas, Paul D. P. Pharoah, Douglas F. Easton, Georgia Chenevix-Trench, Marjanka K. Schmidt

**Affiliations:** 1 Division of Molecular Pathology, The Netherlands Cancer Institute - Antoni van Leeuwenhoek Hospital, Amsterdam, 1066 CX The Netherlands; 2 Department of Genetics and Computational Biology, QIMR Berghofer Medical Research Institute, Brisbane, Queensland Australia; 3 Division of Molecular Carcinogenesis, The Netherlands Cancer Institute - Antoni van Leeuwenhoek Hospital, Amsterdam, The Netherlands; 4 Department of Health and Human Services, Division of Cancer Epidemiology and Genetics, National Cancer Institute, National Institutes of Health, Bethesda, MD USA; 5 Lunenfeld-Tanenbaum Research Institute of Mount Sinai Hospital, Fred A. Litwin Center for Cancer Genetics, Toronto, ON Canada; 6 Department of Molecular Genetics, University of Toronto, Toronto, ON Canada; 7 Department of Medicine, Genetic Epidemiology Research Institute, University of California Irvine, Irvine, CA USA; 8 Division of Clinical Epidemiology and Aging Research, German Cancer Research Center (DKFZ), Heidelberg, Germany; 9 Cancer Prevention Program, Fred Hutchinson Cancer Research Center, Seattle, WA USA; 10 Zilber School of Public Health, University of Wisconsin-Milwaukee, Milwaukee, WI USA; 11 Department of Cancer Epidemiology, Clinical Sciences, Lund University, Lund, Sweden; 12 Institute of Medical Biometry and Epidemiology, University Medical Center Hamburg-Eppendorf, Hamburg, Germany; 13 Department of Gynecology and Obstetrics, Comprehensive Cancer Center Erlangen-EMN, University Hospital Erlangen, Friedrich-Alexander University Erlangen-Nuremberg (FAU), Erlangen, Germany; 14 Division of Cancer Epidemiology, German Cancer Research Center (DKFZ), Heidelberg, Germany; 15 Copenhagen University Hospital, Copenhagen General Population Study, Herlev and Gentofte Hospital, Herlev, Denmark; 16 Department of Clinical Biochemistry, Herlev and Gentofte Hospital, Copenhagen University Hospital, Herlev, Denmark; 17 Faculty of Health and Medical Sciences, University of Copenhagen, Copenhagen, Denmark; 18 Department of Public Health and Primary Care, University of Cambridge, Centre for Cancer Genetic Epidemiology, Cambridge, UK; 19 Division of Preventive Oncology, German Cancer Research Center (DKFZ) and National Center for Tumor Diseases (NCT), Heidelberg, Germany; 20 German Cancer Research Center (DKFZ), German Cancer Consortium (DKTK), Heidelberg, Germany; 21 Institute of the Ruhr University Bochum (IPA), Institute for Prevention and Occupational Medicine of the German Social Accident Insurance, Bochum, Germany; 22 Department of Medicine, Huntsman Cancer Institute, Salt Lake City, UT USA; 23 Division of Research, Kaiser Permanente, Oakland, CA USA; 24 Department of Biology, University of Pisa, Pisa, Italy; 25 German Cancer Research Center (DKFZ), Genomic Epidemiology Group, Heidelberg, Germany; 26 Instituto de Investigacion Sanitaria Galicia Sur (IISGS), Xerencia de Xestion Integrada de Vigo-SERGAS, Oncology and Genetics Unit, Vigo, Spain; 27 Cancer Epidemiology Group, University Cancer Center Hamburg (UCCH), University Medical Center Hamburg-Eppendorf, Hamburg, Germany; 28 Division of Cancer Prevention and Control, Roswell Park Cancer Institute, Buffalo, NY USA; 29 Westmead Institute for Medical Research, University of Sydney, Sydney, New South Wales Australia; 30 Department of Cancer Genetics, Institute for Cancer Research, Oslo University Hospital-Radiumhospitalet, Oslo, Norway; 31 Faculty of Medicine, Institute of Clinical Medicine, University of Oslo, Oslo, Norway; 32 Department of Research, Vestre Viken Hospital, Drammen, Norway; 33 Section for Breast- and Endocrine Surgery, Department of Cancer, Division of Surgery, Cancer and Transplantation Medicine, Oslo University Hospital-Ullevål, Oslo, Norway; 34 Department of Radiology and Nuclear Medicine, Oslo University Hospital, Oslo, Norway; 35 Department of Pathology, Akershus University Hospital, Lørenskog, Norway; 36 Department of Tumor Biology, Institute for Cancer Research, Oslo University Hospital, Oslo, Norway; 37 Department of Oncology, Division of Surgery, Cancer and Transplantation Medicine, Oslo University Hospital-Radiumhospitalet, Oslo, Norway; 38 National Advisory Unit on Late Effects after Cancer Treatment, Oslo University Hospital-Radiumhospitalet, Oslo, Norway; 39 Department of Oncology, Akershus University Hospital, Lørenskog, Norway; 40 Oslo University Hospital, Breast Cancer Research Consortium, Oslo, Norway; 41 Department of Medical Genetics, Oslo University Hospital and University of Oslo, Oslo, Norway; 42 Department of Laboratory Medicine and Pathology, Mayo Clinic, Rochester, MN USA; 43 Department of Oncology and Metabolism, University of Sheffield, Sheffield Institute for Nucleic Acids (SInFoNiA), Sheffield, UK; 44 Academic Unit of Pathology, Department of Neuroscience, University of Sheffield, Sheffield, UK; 45 Department of Medical Epidemiology and Biostatistics, Karolinska Institutet, Stockholm, Sweden; 46 Department of Clinical Genetics, Fox Chase Cancer Center, Philadelphia, PA USA; 47 Gynaecology Research Unit, Hannover Medical School, Hannover, Germany; 48 Nutrition and Metabolism Section, International Agency for Research on Cancer (IARC-WHO), Lyon, France; 49 Department of Oncology, University of Cambridge, Centre for Cancer Genetic Epidemiology, Cambridge, UK; 50 School of Life Sciences, University of Westminster, London, UK; 51 Faculty of Medicine, University of Southampton, Southampton, UK; 52 Institute of Human Genetics, Comprehensive Cancer Center Erlangen-EMN, University Hospital Erlangen, Friedrich-Alexander University Erlangen-Nuremberg (FAU), Erlangen, Germany; 53 Department of Medicine, Brigham and Women’s Hospital and Harvard Medical School, Channing Division of Network Medicine, Boston, MA USA; 54 Department of Epidemiology, Harvard T.H. Chan School of Public Health, Boston, MA USA; 55 Division of Evolution and Genomic Sciences, School of Biological Sciences, Faculty of Biology, Medicine and Health, University of Manchester, Manchester Academic Health Science Centre, Manchester, UK; 56 St Mary’s Hospital, Manchester University NHS Foundation Trust, Manchester Academic Health Science Centre, North West Genomics Laboratory Hub, Manchester Centre for Genomic Medicine, Manchester, UK; 57 Department of Medicine Division of Hematology and Oncology, University of California at Los Angeles, David Geffen School of Medicine, Los Angeles, CA USA; 58 Department of Breast Surgery, Herlev and Gentofte Hospital, Copenhagen University Hospital, Herlev, Denmark; 59 School of Public Health, Curtin University, Perth, Western Australia Australia; 60 Galician Public Foundation of Genomic Medicine (FPGMX), Genomic Medicine Group, International Cancer Genetics and Epidemiology Group, Health Research Institute of Santiago (IDIS), Santiago de Compostela, Spain; 61 University of California San Diego, Moores Cancer Center, La Jolla, CA USA; 62 Instituto de Investigación Sanitaria San Carlos (IdISSC), Centro Investigación Biomédica en Red de Cáncer (CIBERONC), Medical Oncology Department, Hospital Clínico San Carlos, Madrid, Spain; 63 Cancer Epidemiology Division, Cancer Council Victoria, Melbourne, Victoria Australia; 64 Centre for Epidemiology and Biostatistics, Melbourne School of Population and Global Health, The University of Melbourne, Melbourne, Victoria Australia; 65 Precision Medicine, School of Clinical Sciences at Monash Health, Monash University, Clayton, Victoria Australia; 66 Department of Surgery, Oulu University Hospital, University of Oulu, Oulu, Finland; 67 Team Exposome and Heredity, INSERM, University Paris-Saclay, Center for Research in Epidemiology and Population Health (CESP), Villejuif, France; 68 Molecular Epidemiology Group, C080, German Cancer Research Center (DKFZ), Heidelberg, Germany; 69 Molecular Biology of Breast Cancer, University Womens Clinic Heidelberg, University of Heidelberg, Heidelberg, Germany; 70 German Research Center for Environmental Health, Institute of Diabetes Research, Helmholtz Zentrum München, Neuherberg, Germany; 71 Faculty of Medicine and University Hospital Cologne, Center for Familial Breast and Ovarian Cancer, University of Cologne, Cologne, Germany; 72 Faculty of Medicine and University Hospital Cologne, Center for Integrated Oncology (CIO), University of Cologne, Cologne, Germany; 73 Department of Preventive Medicine, Keck School of Medicine, University of Southern California, Los Angeles, CA USA; 74 Institute of Environmental Medicine, Karolinska Institutet, Stockholm, Sweden; 75 Department of Oncology, Södersjukhuset, Stockholm, Sweden; 76 Molecular Genetics of Breast Cancer, German Cancer Research Center (DKFZ), Heidelberg, Germany; 77 Department of Health Sciences Research, Mayo Clinic, Rochester, MN USA; 78 Translational Cancer Research Area, University of Eastern Finland, Kuopio, Finland; 79 Institute of Clinical Medicine, Pathology and Forensic Medicine, University of Eastern Finland, Kuopio, Finland; 80 Institute of Pathology, Comprehensive Cancer Center Erlangen Nuremberg, University Hospital Erlangen, Friedrich-Alexander-University Erlangen-Nuremberg, Erlangen, Germany; 81 Department of Medical Oncology, Erasmus MC Cancer Institute, Rotterdam, The Netherlands; 82 Dr. Margarete Fischer-Bosch-Institute of Clinical Pharmacology, Stuttgart, Germany; 83 University of Tübingen, Tübingen, Germany; 84 Division of Cancer Sciences, University of Manchester, Manchester, UK; 85 Nuffield Department of Population Health, University of Oxford, Oxford, UK; 86 Westmead Institute for Medical Research, Australian Breast Cancer Tissue Bank, University of Sydney, Sydney, New South Wales Australia; 87 Research Department, Peter MacCallum Cancer Center, Melbourne, Victoria Australia; 88 Sir Peter MacCallum Department of Oncology, The University of Melbourne, Melbourne, Victoria Australia; 89 Department of Genetics and Pathology, Pomeranian Medical University, Szczecin, Poland; 90 Independent Laboratory of Molecular Biology and Genetic Diagnostics, Pomeranian Medical University, Szczecin, Poland; 91 Department of Gynaecology and Obstetrics, University Hospital Ulm, Ulm, Germany; 92 Department of Epidemiology & Population Health, Stanford University School of Medicine, Stanford, CA USA; 93 Department of Medicine, Division of Oncology, Stanford Cancer Institute, Stanford University School of Medicine, Stanford, CA USA; 94 Department of Radiation Oncology, University Hospitals Leuven, , University of Leuven, Leuven, Belgium; 95 Division of Cancer Epidemiology and Genetics, Radiation Epidemiology Branch, National Cancer Institute, Bethesda, MD USA; 96 Program in Genetic Epidemiology and Statistical Genetics, Harvard T.H. Chan School of Public Health, Boston, MA USA; 97 Department of Computational and Quantitative Medicine, City of Hope, Duarte, CA USA; 98 City of Hope Comprehensive Cancer Center, City of Hope, Duarte, CA USA; 99 VIB Center for Cancer Biology, Leuven, Belgium; 100 Laboratory for Translational Genetics, Department of Human Genetics, University of Leuven, Leuven, Belgium; 101 Epidemiology Program, University of Hawaii Cancer Center, Honolulu, HI USA; 102 Department of Molecular Medicine and Surgery, Karolinska Institutet, Stockholm, Sweden; 103 Department of Clinical Genetics, Karolinska University Hospital, Stockholm, Sweden; 104 Clinical Gerontology, Department of Public Health and Primary Care, University of Cambridge, Cambridge, UK; 105 Kuopio University Hospital, Biobank of Eastern Finland, Kuopio, Finland; 106 Department of Clinical Science and Education, Karolinska Institutet, Södersjukhuset, Stockholm, Sweden; 107 Department of Family Medicine and Public Health, University of California San Diego, La Jolla, CA USA; 108 Department of Medical Oncology, University Hospital of Heraklion, Heraklion, Greece; 109 Biostatistics Unit, The Cyprus Institute of Neurology & Genetics, Nicosia, Cyprus; 110 The Cyprus Institute of Neurology & Genetics, Cyprus School of Molecular Medicine, Nicosia, Cyprus; 111 Department of Laboratory Medicine and Pathobiology, University of Toronto, Toronto, ON Canada; 112 University Health Network, Laboratory Medicine Program, Toronto, ON Canada; 113 Department of Obstetrics and Gynecology, Helsinki University Hospital, University of Helsinki, Helsinki, Finland; 114 Department of Epidemiology, Gillings School of Global Public Health and UNC Lineberger Comprehensive Cancer Center, University of North Carolina at Chapel Hill, Chapel Hill, NC USA; 115 Centre for Cancer Research and Cell Biology, Queen’s University Belfast, Belfast, Northern Ireland, UK; 116 Department of Population Science, American Cancer Society, Atlanta, GA USA; 117 Unit of Medical Genetics, Department of Medical Oncology and Hematology, Fondazione IRCCS Istituto Nazionale dei Tumori di Milano, Milan, Italy; 118 Genome Diagnostics Program, IFOM - the FIRC Institute of Molecular Oncology, Milan, Italy; 119 MASA, Research Centre for Genetic Engineering and Biotechnology ‘Georgi D. Efremov’, Skopje, Republic of North Macedonia; 120 Carmel Medical Center and Technion Faculty of Medicine, Clalit National Cancer Control Center, Haifa, Israel; 121 Medical Oncology Department, Hospital Universitario Puerta de Hierro, Madrid, Spain; 122 Department of Oncology, UCLH Foundation Trust, London, UK; 123 Department of Oncology, University Hospital of Larissa, Larissa, Greece; 124 School of Cancer & Pharmaceutical Sciences, Comprehensive Cancer Centre, Guy’s Campus, King’s College London, London, UK; 125 Faculty of Medicine and University Hospital Cologne, University of Cologne, Center for Molecular Medicine Cologne (CMMC), Cologne, Germany; 126 University Hospital and German Cancer Research Center, National Center for Tumor Diseases, Heidelberg, Germany; 127 Medical Faculty, University Clinic of Radiotherapy and Oncology, Ss. Cyril and Methodius University in Skopje, Skopje, Republic of North Macedonia; 128 Department of Clinical Pathology, The University of Melbourne, Melbourne, Victoria Australia; 129 Genetic Epidemiology Group, School of Population and Global Health, University of Western Australia, Perth, Western Australia Australia; 130 Division of Genetics and Epidemiology, The Institute of Cancer Research, London, UK; 131 Division of Breast Cancer Research, The Institute of Cancer Research, London, UK; 132 Department of Population Health Sciences, Weill Cornell Medicine, New York, NY USA; 133 Department of Epidemiology, Mailman School of Public Health, Columbia University, New York, NY USA; 134 Department of Surgery, Leiden University Medical Center, Leiden, The Netherlands; 135 Institute of Cancer and Genomic Sciences, University of Birmingham, Birmingham, UK; 136 University of Oxford, Wellcome Trust Centre for Human Genetics and Oxford NIHR Biomedical Research Centre, Oxford, UK; 137 Department of Health Science Research, Division of Epidemiology, Mayo Clinic, Rochester, MN USA; 138 Laboratory of Cancer Genetics and Tumor Biology, Cancer and Translational Medicine Research Unit, Biocenter Oulu, University of Oulu, Oulu, Finland; 139 Laboratory of Cancer Genetics and Tumor Biology, Northern Finland Laboratory Centre Oulu, Oulu, Finland; 140 Department of Surgical Sciences, Uppsala University, Uppsala, Sweden; 141 iFIT-Cluster of Excellence, University of Tübingen, Tübingen, Germany; 142 German Cancer Research Center (DKFZ) and German Cancer Consortium (DKTK) Partner Site Tübingen, Tübingen, Germany; 143 Division of Psychosocial Research and Epidemiology, The Netherlands Cancer Institute - Antoni van Leeuwenhoek Hospital, Amsterdam, The Netherlands

**Keywords:** Common germline genetic variants, Breast cancer-specific survival, Patient subgroups, Tumor biology, Systemic treatment

## Abstract

**Background:**

Given the high heterogeneity among breast tumors, associations between common germline genetic variants and survival that may exist within specific subgroups could go undetected in an unstratified set of breast cancer patients.

**Methods:**

We performed genome-wide association analyses within 15 subgroups of breast cancer patients based on prognostic factors, including hormone receptors, tumor grade, age, and type of systemic treatment. Analyses were based on 91,686 female patients of European ancestry from the Breast Cancer Association Consortium, including 7531 breast cancer-specific deaths over a median follow-up of 8.1 years. Cox regression was used to assess associations of common germline variants with 15-year and 5-year breast cancer-specific survival. We assessed the probability of these associations being true positives via the Bayesian false discovery probability (BFDP < 0.15).

**Results:**

Evidence of associations with breast cancer-specific survival was observed in three patient subgroups, with variant rs5934618 in patients with grade 3 tumors (15-year-hazard ratio (HR) [95% confidence interval (CI)] 1.32 [1.20, 1.45], P = 1.4E−08, BFDP = 0.01, per G allele); variant rs4679741 in patients with ER-positive tumors treated with endocrine therapy (15-year-HR [95% CI] 1.18 [1.11, 1.26], P = 1.6E−07, BFDP = 0.09, per G allele); variants rs1106333 (15-year-HR [95% CI] 1.68 [1.39,2.03], P = 5.6E−08, BFDP = 0.12, per A allele) and rs78754389 (5-year-HR [95% CI] 1.79 [1.46,2.20], P = 1.7E−08, BFDP = 0.07, per A allele), in patients with ER-negative tumors treated with chemotherapy.

**Conclusions:**

We found evidence of four loci associated with breast cancer-specific survival within three patient subgroups. There was limited evidence for the existence of associations in other patient subgroups. However, the power for many subgroups is limited due to the low number of events. Even so, our results suggest that the impact of common germline genetic variants on breast cancer-specific survival might be limited.

**Supplementary Information:**

The online version contains supplementary material available at 10.1186/s13058-021-01450-7.

## Introduction

Inherited common genetic variation is likely to influence survival in breast cancer patients [1]. Results from pre-clinical experiments have shown different metastatic behaviors in mice with different genetic backgrounds [2–7]. In addition, familial studies of breast cancer patients have shown that women with a first-degree relative with a poor prognosis breast cancer have a worse prognosis compared to women with a first-degree relative with a good prognosis cancer [8]. Moreover, genome-wide and candidate gene association studies have discovered common genetic variants associated with specific subtypes of breast cancer based on the expression of the estrogen receptor (ER) [9–11], progesterone receptor (PR), and the amplification of the human epidermal growth factor receptor 2 (HER2) [12, 13], which are known breast cancer prognostic factors [14, 15]. Finally, a number of studies have suggested that specific common germline genetic variants affect breast cancer prognosis both overall and within subgroups of patients [16–24].

Despite the supporting evidence, it remains challenging to identify common germline variants associated with breast cancer-specific survival. This may partially be explained by the good prognosis of breast cancer patients, which leads to underpowered analyses. Even large studies based on worldwide consortia cannot reach the number of breast cancer deaths necessary to detect small to moderate associations at a genome-wide significant level [19, 20, 25]. However, breast cancer is a heterogeneous disease, and it is possible that stronger associations between common germline variants and breast cancer-specific survival are present in certain patient subgroups, but cannot be detected in breast cancer overall. Previous studies provide modest evidence supporting this hypothesis [16, 19, 20].

The aim of our study was to evaluate the evidence for associations of inherited common genetic variants with breast cancer-specific survival within more homogeneous subgroups of breast cancer patients, defined by prognostic factors representative of tumor biology and/or by the type of systemic treatment. To this end, we performed genome-wide association analyses within clinically relevant, defined subgroups of patients based on hormone receptors, tumor grade, age at diagnosis, and type of systemic treatment [26, 27]. We also explored the subgroup-specific associations identified by previous studies [16, 19, 23, 28, 29], to confirm or refute those results.

## Materials and methods

### Study sample

We selected female breast cancer patients of European ancestry from studies participating in the Breast Cancer Association Consortium (BCAC). We included patients with available information about vital status and number of years from diagnosis to last follow-up who were diagnosed with a primary invasive breast cancer of any stage and were at least 18 years old at diagnosis. The final study sample consisted of 91,686 breast cancer patients from 70 BCAC studies. A description of the included studies is given in Additional file 1: Supplementary Table S1.

Information about histopathology, survival, and treatment was collected by individual studies and pooled and harmonized at the Netherlands Cancer Institute before incorporation into the BCAC database at the University of Cambridge (version 12, July 2019). All studies were approved by the relevant ethics committees and informed consent was obtained from all patients.

### Patient subgroups

The subgroups of interest were defined based on age at diagnosis, estrogen receptor (ER) status, progesterone receptor (PR) status, human epidermal growth factor receptor 2 (HER2) status, tumor grade, and the use and type of systemic treatment, as available in the BCAC database. For age at diagnosis and tumor grade, we focused on subgroups characterized by worse prognosis. We thus defined 15 subgroups: (a) patients younger than age 40 years at diagnosis; (b) patients with grade 3 tumors; (c) patients with ER-positive (ER+) tumors, who received endocrine therapy (any kind); (d) patients diagnosed with ER-negative (ER−) tumors, who received chemotherapy (any kind); (e) patients with tumors that were hormone receptor (HR) positive (ER+ or PR+) and HER2-negative (HER2−); (f) patients with HR-positive (HR+), HER2− tumors, who received chemotherapy (any kind); (g) patients with HR+, HER2− tumors, who did not receive chemotherapy; (h) patients with HR+, HER2-positive (HER2+) tumors; (i) patients with HR-negative (HR−), HER2+ tumors, (j) patients with HR−, HER2− tumors; (k) patients who received Tamoxifen; (l) patients who received an aromatase inhibitor; (m) patients who received a Cyclophosphamide Methotrexate Fluorouracil (CMF)-like chemotherapy regimen; (n) patients who received taxanes; (o) patients who received anthracyclines.

The rationale and references to the literature supporting the choice of each subgroup for inclusion in a genome-wide association study on survival are given in Additional file 1: Supplementary Table S2. We did not include the subgroup of HER2+ tumors treated with Trastuzumab because of a relatively small number of patients and low event rate, leading to analyses that are more underpowered than those presented.

Patients with metastatic breast tumors at diagnosis (1.1 % of all included patients) were excluded from the subgroup analyses whose definition was based on the use and type of systemic therapy as generally they are treated with palliative intent [15, 30, 31].

In addition to the subgroup analyses, we also performed a genome-wide analysis of 15-year breast cancer-specific survival in all breast cancer patients. We performed this analysis to evaluate whether associations between common germline variants and breast cancer-specific survival in subgroups could be detected in the full dataset of patients. Genome-wide analyses for survival (unstratified by subtype) were previously performed [20] based on 12 GWAS datasets, but these included fewer patients from iCOGS and OncoArray (n = 84,757), with shorter follow-up, than were available in the current dataset. We focused our analyses on the iCOGS and OncoArray datasets, because the remaining 10 GWAS datasets used in the previous study did not include information about tumor characteristics, beyond ER status, or treatment, which were crucial for the subgroup analyses.

Due to the presence of missing values in the variables used to define the subgroups, not all patients could be classified by each subgroup. The number of patients included in each subgroup, together with the number of breast cancer-specific deaths, patient/tumor characteristics, treatment, and follow-up information, are shown in Additional file 1: Supplementary Tables S3-S4, and Additional file 2: Supplementary Table S5.

### Imputation of missing values in clinical and pathological variables

For secondary adjusted analyses, we imputed missing values in the clinical and pathological variables using the Multiple imputation by Chained Equations (MICE) R package (v. 3.2.0), as described in Additional file 2: Supplementary Methods. A list of imputed variables and corresponding percentages of missing values and imputation methods is provided in Additional file 2: Supplementary Table S6.

### Genotyping and imputation of genetic variants, ancestry analysis, and quality controls

Methods related to genotyping and genotype imputation have been described previously [17, 18, 20]. In brief, patients were genotyped with two different arrays: iCOGS and OncoArray [17, 32]. Only samples that were inferred to have European ancestry, based on genotype data, were included in the analyses. Non-genotyped variants were initially imputed based on the 1000 Genomes Project Phase 3 (October 2014) release as reference panel. More recently, non-genotyped single-nucleotide variants (SNVs) were re-imputed using a reference panel from the Haplotype Reference Consortium (HRC) [33] in order to improve imputation quality, especially for rarer variants. Analyses were performed on genotyped variants or imputed variants with a minor allele frequency (MAF) > 0.01. Imputed variants were included in the analyses if they had imputation *r*^2^ > 0.7. Approximately 10 million variants were analyzed.

### Statistical analyses

The outcome in the analyses was breast cancer-specific survival (time to death due to breast cancer). Hazard ratios (HR) and 95% confidence intervals (CI) were estimated using delayed entry Cox regression models, where the time at risk was considered as starting from the time of study entry if the study entry was after diagnosis (22.9% within 1 year after diagnosis, and 27.3% more than 1 year after diagnosis) and from diagnosis if the time of study entry was missing (24.5%), at diagnosis (16.9%) or before diagnosis (8.4%). The time-to-event was right censored at the time of last follow-up, or at 15 years after diagnosis, whichever came first. Patients who died of unknown cause or causes other than breast cancer were censored at the time of death if death occurred before 15 years from diagnosis or at 15 years otherwise.

With reference to the results of Early Breast Cancer Trialists’ Collaborative Group (EBCTCG) [34], we additionally performed analyses within the subgroups whose definition was based on the use and type of systemic therapy, where we restricted the maximum follow-up time to 5 years after diagnosis (Additional file 2: Supplementary Table S5). The goal of those analyses was to investigate the potential short-term effects of germline variants on patients who received specific types of systemic treatment, since the effect might not be constant over time and treatment plans tend to focus on the first 5 years after diagnosis [15, 31].

**Fig. 1:**
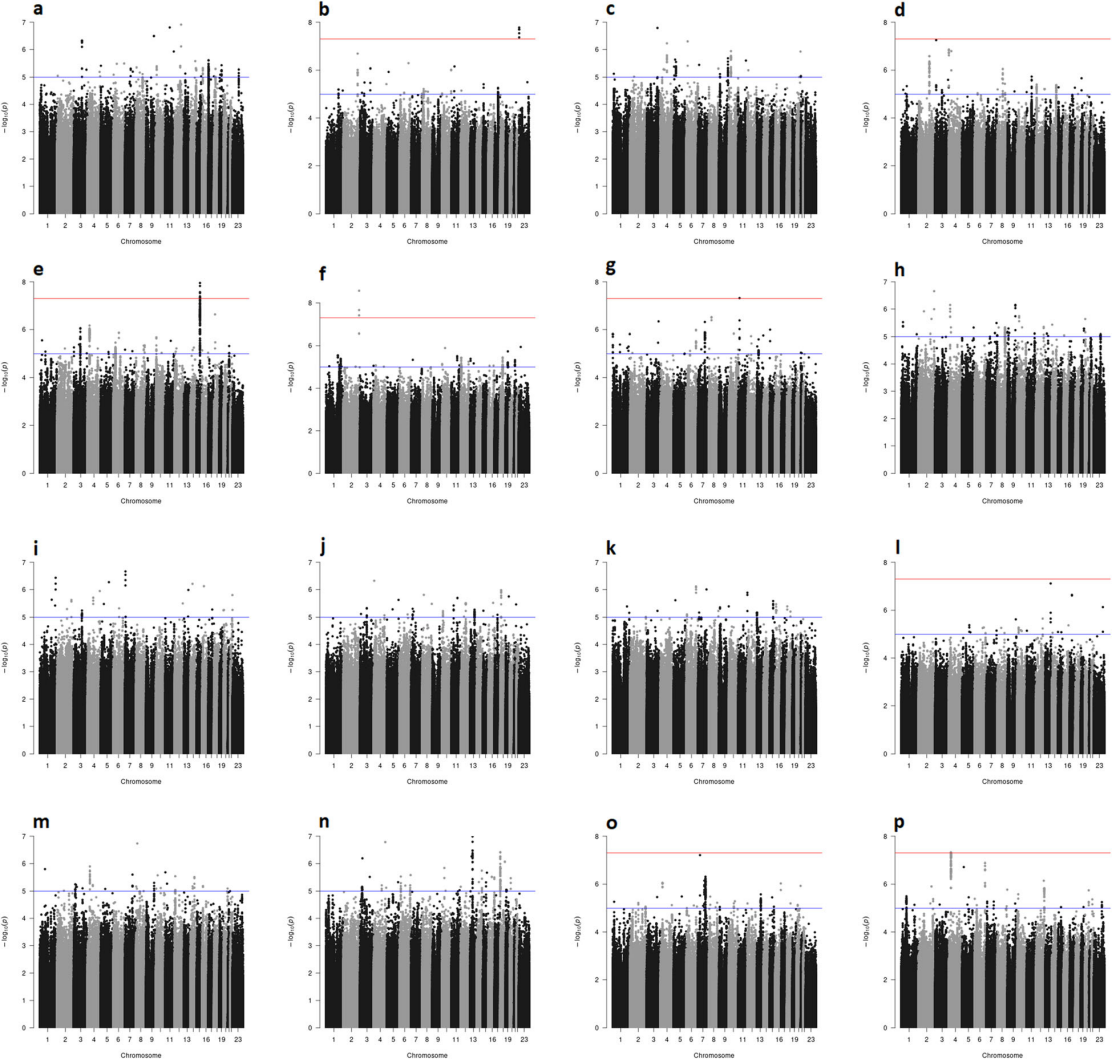
Genome-wide analyses of 15-year breast cancer-specific survival. The Manhattan plots show the results of the unadjusted analyses (stratified by country). Results are represented as −log10 of the p value from Cox regression models (y-axis). The x-axis shows the chromosome number, where chromosome 23 represents the X chromosome. The red line represents the genome-wide significant threshold 5E−08, the blue line corresponds to the threshold 1E−05. Breast cancer patients included in the analysis in panel: **a** younger than age 40 years at diagnosis; **b** diagnosed with a grade 3 tumor; **c** diagnosed with an ER+ tumor and treated with (any) endocrine therapy; **d** diagnosed with a ER**−** tumor and treated with (any) chemotherapy; **e** diagnosed with an ER+ or PR+, and HER2**−** tumor; **f** diagnosed with an ER+ or PR+, and HER2**−** tumor treated with (any) chemotherapy; **g** diagnosed with an ER+ or PR+, and HER2**−** tumor not treated with chemotherapy; **h** diagnosed with an ER+ or PR+, and HER2+ tumor; **i** diagnosed with an ER**−** and PR**−** and HER2+ tumor; **j** diagnosed with an ER**−** and PR**−** and HER2**−** tumor; **k** treated with tamoxifen; **l** treated with aromatase inhibitor; **m** treated with CMF-like chemotherapy; **n** treated with taxanes; **o** treated with anthracyclines; **p** all

**Fig. 2:**
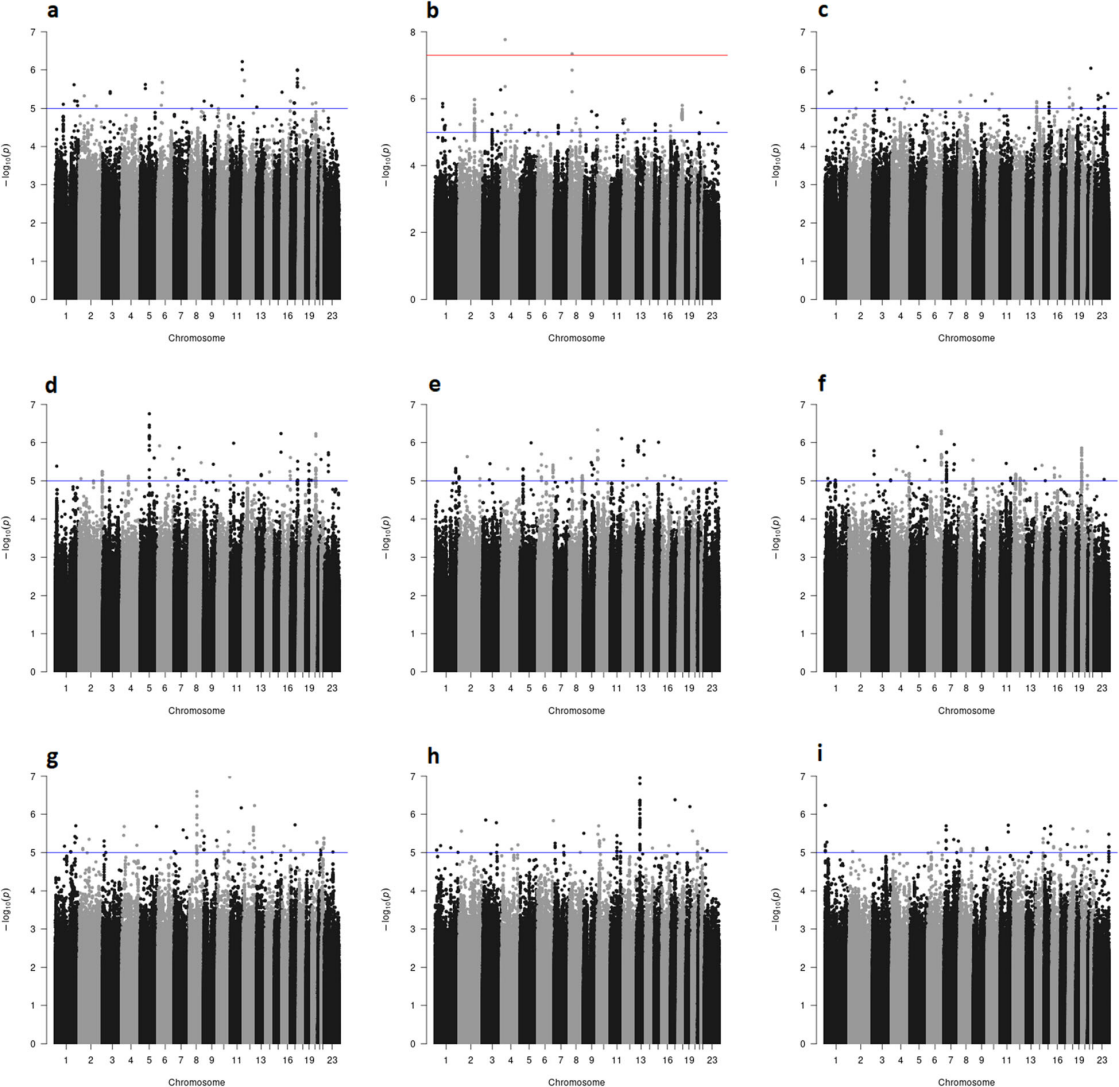
Genome-wide analyses of 5-year breast cancer-specific survival. The Manhattan plots show the results of the unadjusted analyses (stratified by country). Results are represented as −log10 of the p value from Cox regression models (y-axis). The x-axis shows the chromosome number, where chromosome 23 represents the X chromosome. The red line represents the genome-wide significant threshold 5E−08, the blue line corresponds to the threshold 1E−05. Breast cancer patients included in the analysis in panel: **a** diagnosed with a ER+ tumor and treated with (any) endocrine therapy; **b** diagnosed with a ER− tumor and treated with (any) chemotherapy; **c** diagnosed with a ER+ or PR+, and HER2− tumor treated with (any) chemotherapy; **d** diagnosed with a ER+ or PR+, and HER2− tumor not treated with chemotherapy; **e** treated with tamoxifen; **f** treated with aromatase inhibitor; **g** treated with CMF-like chemotherapy; **h** treated with taxanes; **i** treated with anthracyclines

Cox regression analysis was performed within each subgroup of interest, separately, and was stratified by country. All the analyses were performed separately by genotyping platform (iCOGS vs OncoArray), and the results were combined via a fixed-effects meta-analysis. The standard errors of the HR estimates were re-computed based on the likelihood ratio test statistic, as done previously [20] (Figs. 1 and 2). For variants that satisfied the inclusion criteria (MAF>0.01 and *r*^2^ > 0.7) on only one genotyping platform, we included the result for that specific platform (Tables 1 and 2). However, for variants with an association P < 5E−08, we also computed HR and 95% confidence interval in the other genotyping platform to verify that the direction of the association was the same (Additional file 2: Supplementary Table S7).

**Table 1:** GWAS significant (P<5×10-8) and noteworthy (BFDP<0.15) results by subgroup, and corresponding results from adjusted analyses

Subgroup	Variant	Chr	Position	Alleles^a^	AAF	Unadjusted analyses			Adjusted analyses		
						HR [95% CI]	*P* value	BFDP	HR [95% CI]	*P* value	BFDP
Grade 3 tumors	rs5934618^b^	X	9437463	A/G	0.08	1.39 [1.24,1.56]	1.7E-08	0.02	1.36 [1.21,1.53]^e^	3.0E-07	0.17
	rs4830644^b^	X	9434808	A/G	0.08	1.39 [1.24,1.56]	2.0E-08	0.02	1.35 [1.20,1.52]^e^	4.8E-07	0.23
	rs3810742^b, c^	X	9432603	T/C	0.08	1.38 [1.24,1.55]	2.0E-08	0.02	1.35 [1.20,1.52]^e^	4.2E-07	0.20
	rs4830642^b^	X	9431786	T/C	0.08	1.38 [1.24,1.55]	2.9E-08	0.02	1.35 [1.20,1.52]^e^	5.8E-07	0.26
	rs72611496^b^	X	9434264	G/A	0.08	1.38 [1.24,1.55]	4.3E-08	0.03	1.34 [1.19,1.51]^e^	1.2E-06	0.40
	rs66871326	2	209048052	AAGGAG/A	0.76	0.85 [0.80,0.90]	2.1E-07	0.11	0.86 [0.81,0.92]^e^	1.8E-06	0.49
ER+ or PR+, and HER2-	rs8030394	15	71637241	C/T	0.99	2.47 [1.81,3.37]	1.1E-08	0.42	2.38 [1.74,3.27]^f^	7.6E-08	0.72
	rs112641969	15	71715016	A/G	0.02	0.46 [0.35,0.61]	4.6E-08	0.46	0.48 [0.36,0.64]^f^	3.7E-07	0.78
	rs16955466	15	71637757	C/T	0.01	0.40 [0.29,0.55]	1.5E-08	0.49	0.42 [0.31,0.58]^f^	1.8E-07	0.82
	rs7165279	15	71636591	T/C	0.99	2.41 [1.77,3.28]	2.7E-08	0.54	2.33 [1.70,3.19]^f^	1.4E-07	0.78
	rs111962948	15	71656213	G/T	0.01	0.41 [0.29,0.56]	3.0E-08	0.61	0.43 [0.31,0.60]^f^	5.6E-07	0.91
	rs112813972	15	71577932	T/C	0.02	0.40 [0.28,0.55]	4.0E-08	0.70	0.42 [0.30,0.59]^f^	3.7E-07	0.90
ER+ or PR+, and HER2- treated with CT	rs62192052	2	230372348	C/T	0.02	0.15 [0.08, 0.28]	2.6E-09	0.99	0.15 [0.08, 0.29]^g^	5.5E-09	0.99
	rs74423556^c^	2	230325234	C/G	0.02	0.16 [0.08,0.30]	2.1E-08	0.99	0.16 [0.08,0.31]^g^	3.8E-08	1.00
	rs145983608	2	230296944	A/G	0.02	0.15 [0.08,0.30]	3.8E-08	1.00	0.15 [0.08,0.31]^g^	1.1E-07	1.00
ER+ or PR+, and HER2- not treated with CT	rs56248395^b^	11	20084391	C/T	0.13	2.33 [1.72,3.15]	4.8E-08	0.59	2.23 [1.66,2.99]^g^	1.2E-07	0.69
**ER+ treated with ET**	**rs4679741**	**3**	**155003603**	**T/G**	**0.49**	**1.18 [1.11,1.26]**	**1.6E-07**	**0.09**	**1.20 [1.13,1.28]**^**h**^	**1.1E-08**	**0.01**
**ER- treated with CT**	**rs78754389**^**d**^	**4**	**35962454**	**G/A**	**0.07**	**1.79 [1.46,2.20]**	**1.7E-08**	**0.07**	**1.67 [1.39,2.00]**^**i**^	**4.1E-08**	**0.09**
	**rs1106333**	**3**	**14562127**	**C/A**	**0.06**	**1.68 [1.39,2.03]**	**5.6E-08**	**0.12**	**1.70 [1.41,2.05]**^**i**^	**4.4E-08**	**0.11**
	rs117685664^d^	8	26989084	C/T	0.03	0.26 [0.16,0.42]	4.6E-08	0.97	0.50 [0.35,0.70]^i^	6.4E-05	0.99
Tamoxifen	rs72775397^d^	5	94266932	C/T	0.28	1.36 [1.21,1.53]	1.8E-07	0.11	1.11 [1.03,1.19]^j^	6.6E-03	1.00
Anthracylines	rs34072391	7	30243729	C/CA	0.52	1.27 [1.17,1.39]	6.2E-08	0.04	1.26 [1.15,1.37]^k^	3.4E-07	0.16

*Abbreviations*: *Chr* chromosome, *ER* estrogen receptor, *PR* progesterone receptor, *HER2* human epidermal growth factor receptor 2, *ET* Endocrine therapy, *CT* chemotherapy, *AAF* alternative allele frequency, *HR* hazard ratio, *CI* confidence interval, *BFDP* Bayesian False Discovery Probability

Note: BFDP is computed assuming the prior probability of true association equal to 10^-4^for all variants, which implies a number of expected true associations in the order of 10^2^. Results with BFDP<0.15 in the adjusted analyses are bolded. ^**a**^Reference/Alternative alleles, ^b^Analyses only include OncoArray data since the variants had imputation r^2^ <0.7 on iCOGS. More detailed analyses are reported in Table 2 and Supplementary Table 7, ^**c**^Variant genotyped on OncoArray, ^d^From the 5-years breast cancer specific survival analysis, ^e^Adjusted for age at diagnosis, lymph node status, tumor size, distant metastases status, ER status, HER2 status, (neo)adjuvant CT, ^f^Adjusted for age at diagnosis, lymph node status, tumor size, tumor grade, distant metastases status, and (neo)adjuvant CT, ^g^Adjusted for age at diagnosis, lymph node status, tumor size, and tumor grade, ^h^Adjusted for age at diagnosis, lymph node status, tumor size, tumor grade, HER2 status, (neo)adjuvant CT, ^i^Adjusted for age at diagnosis, lymph node status, tumor size, tumor grade, and HER2 status, ^j^Adjusted for age at diagnosis, lymph node status, tumor size, tumor grade, HER2 status ,and (neo)adjuvant CT, ^k^Adjusted for age at diagnosis, lymph node status, tumor size, tumor grade, ER status, and HER2 status

**Table 2:** Meta-analysis results for variants analysed on OncoArray only in the unadjusted analyses in Table 1

Subgroup	Variant	Chr	Position	Alleles^a^	AAF	Unadjusted meta-analysis			Adjusted meta-analysis		
						HR [95% CI]	*P* value	BFDP	HR [95% CI]	*P* value	BFDP
**Grade 3 tumors**	**rs5934618**	**X**	**9437463**	**A/G**	**0.08**	**1.32 [1.20,1.45]**	**1.4E-08**	**0.01**	**1.31 [1.18, 1.44]**^**b**^	**7.9E-08**	**0.05**
	rs4830644		9434808	A/G	0.08	1.32 [1.20,1.45]	2.1E-08	0.01	1.30 [1.18, 1.43]^b^	1.7E-07	0.10
	rs3810742		9432603	T/C	0.08	1.31 [1.19,1.44]	2.7E-08	0.02	1.29 [1.17, 1.42]^b^	1.8E-07	0.10
	rs4830642		9431786	T/C	0.08	1.31 [1.19,1.44]	2.8E-08	0.02	1.29 [1.17, 1.42]^b^	2.2E-07	0.12
	rs72611496		9434264	G/A	0.08	1.32 [1.20,1.45]	2.3E-08	0.02	1.30 [1.18, 1.43]^b^	2.5E-07	0.13
ER+ or PR+, and HER2- not treated with CT	rs56248395	11	20084391	C/T	0.13	1.53 [1.25,1.89]	5.2E-05	0.97	1.52 [1.24,1.87]^c^	6.4E-05	0.98

*Abbreviations*: *Chr* chromosome, *AAF* alternative allele frequency, *HR* hazard ratio, *CI* confidence interval, *BFDP* Bayesian False Discovery Probability

Note: BFDP is computed assuming the prior probability of true association equal to 10^-4^ for all variants, which implies a number of expected true associations in the order of 10^2^. Results with BFDP<0.15 in the adjusted analyses are bolded. ^**a**^Reference/Alternative alleles, ^b^Adjusted for age at diagnosis, lymph node status, tumor size, distant metastases status, ER status, HER2 status, (neo)adjuvant CT, ^c^Adjusted for age at diagnosis, lymph node status, tumor size, and tumor grade

Inflation of the likelihood ratio test statistics was estimated, within each subgroup, by dividing the median of the observed test statistics values by the median of a ${\chi}_1^2$ distribution (Additional file 2: Supplementary Figures S1 and S2). To assess the noteworthiness of the observed associations, we made use of the Bayesian false discovery probability (BFDP) measure [35]. To compute BFDPs, we set the prior probability of true association to 10^−4^ [36, 37], as done previously [20], and chose the prior distribution of the log hazard ratio of interest (effect size of a variant) to be a Normal distribution with mean 0 and standard error equal to 0.2 [36]. We describe associations with BFDP < 0.15 as “noteworthy” [20]. For each noteworthy result at a prior of 10^−4^, we also provided BFDPs under two, more restrictive, prior probabilities of true association (10^−5^ and 10^−6^; Additional file 3: Supplementary Tables S8-S9) [36, 37]. In addition, we estimated the power to detect genetic variant associated with 15-year and 5-year breast cancer-specific survival by subgroup (Additional file 3: Supplementary Table S10 and S11) as described in Additional file 2: Supplementary Methods.

For each genome-wide significant (P < 5E−08) [38] and/or noteworthy (BFDP < 0.15) association observed in the primary unadjusted subgroup analyses, we performed secondary analyses adjusted for age at diagnosis, tumor characteristics, and type of systemic treatment not used in the definition of the specific subgroup in which the association was detected (Tables 1 and 2). Secondary adjusted analyses were performed to account for residual heterogeneity; we used imputed covariates in order to keep the same sample size.

For each genome-wide significant or noteworthy association in the primary unadjusted analyses, we looked at the functional annotation of the surrounding genomic area, using the Functional Mapping and Annotation of Genome-Wide Association Studies (FUMA GWAS) tool [39] (Additional file 2: Supplementary Figures S5 and S6). We also tested whether the expression of the nearest genes correlated with distant metastasis-free survival in breast cancer patients using KMplotter [40, 41].

PancanQTL [42] was used to identify cis-expression quantitative trait locus (eQTLs), trans-eQTLs, and survival eQTLs in breast cancer to see whether the genome-wide significant or noteworthy genetic variants from the primary analyses could be linked with the expression levels of genes affecting survival. In addition, for all the genome-wide significant and/or noteworthy associations detected in the primary analyses, we searched the GWAS catalog [43] to see whether there was already evidence of those being associated with breast cancer or other traits.

## Results

After a median follow-up of 8.1 years, there were a total of 7531 breast cancer deaths among 91686 breast cancer patients (Additional file 1: Supplementary Tables S3-S4 and Additional file 2: Supplementary Table S5).

In the 15-year breast cancer-specific survival analyses, power for detecting genome-wide significant associations was < 0.45 for effect sizes (HRs) < 1.20 in all subgroups investigated and for all minor allele frequencies. The power was highest in the subgroups of grade 3 tumors, ER+ tumors treated with endocrine therapy, HR+ and HER2− tumors, and patients who received tamoxifen (Additional file 3: Supplementary Table S10). In the 5-year breast cancer-specific survival analyses, power was highest in the subgroups of patients with an ER− tumor who received chemotherapy, patients who received tamoxifen, and patients who received anthracyclines (Additional file 3: Supplementary Table S11).

Genome-wide significant and/or noteworthy associations with 15-year or 5-year breast cancer-specific survival were observed in the unadjusted analyses based on all patients (Additional file 2: Supplementary Table S7) and analyses of eight out of the 15 subgroups investigated (Tables 1 and 2; Figs. 1 and 2). The genomic inflation factor of the unadjusted genome-wide analyses varied from 0.981 to 1.028 (Additional file 2: Supplementary Figures S1- S4); it is therefore unlikely that the association results were affected by cryptic population substructure.

Two genome-wide significant associations were observed in the unstratified analysis based on patients genotyped using OncoArray (Additional file 2: Supplementary Table S7), namely variants rs57714252 (P = 4.7E−08) and rs4129285 (P = 4.9E−08), both situated in an intergenic region of chromosome 4 (Additional file 2: Supplementary Figure S5). These results were only based on the OncoArray data, since on iCOGS the variants did not satisfy the inclusion criteria for genotypes (iCOGS imputation *r*^2^ = 0.62). The corresponding estimates in the iCOGS data were in the opposite direction compared to the OncoArray estimates, and the results from the meta-analysis were not genome-wide significant and showed a large BFDP (Additional file 2: Supplementary Table S7).

Genome-wide significant associations were observed in the analysis restricted to patients diagnosed with a grade 3 tumor, with five correlated variants (Tables 1 and 2) located on chromosome X (Additional file 2: Supplementary Figure S5) in intron 1 of *TBL1X*. For the most significant variant, rs5934618, the alternative G allele was associated with increased risk of breast cancer death in unadjusted analyses (meta-analysis hazard ratio (HR) [95% confidence interval (CI)] 1.32 [1.20, 1.45], P = 1.4E−08, BFDP = 0.01; Table 2). The meta-analysis result remained substantially unchanged after adjusting for age at diagnosis, additional tumor characteristics, and treatment with (neo)adjuvant chemotherapy (HR [95% CI] 1.31 [1.18, 1.44], P = 7.9E−08, BFDP = 0.05; Table 2). The variant was not associated with the outcome in lower-grade tumors or in all patients combined (heterogeneity by grade P = 1.5E−03; Additional file 2: Supplementary Figure S7). All the five variants overlap chromatin features H3K4me3 and H3K27ac (associated with active transcription start sites) in multiple mammary cell types from normal breast tissue (Additional file 2: Supplementary Figure S8). Furthermore, there was evidence of *TBL1X* expression being associated with distant metastasis-free survival (HR [95%CI] for high vs low expression 1.71 [1.20,2.44], P = 2.7E−03) specifically in grade 3 patients, but not in patients with lower-grade disease (Additional file 2: Supplementary Figure S9). However, there was no evidence of association with *TBL1X* expression in normal breast tissue with any of the variants identified in our genome-wide analyses (Additional file 2: Supplementary Figure S10).

In the same subgroup of grade 3 tumors, we observed a noteworthy, non-genome-wide significant association with variant rs66871326, located on chromosome 2 in an intron of *C2orf80*. For variant rs66871326, the alternative A allele was associated with decreased risk of breast cancer death (HR [95% CI] 0.85 [0.80,0.90], P = 2.1E−07, BFDP = 0.11). The corresponding BFDP increased to 0.49 after adjusting for age at diagnosis, additional tumor characteristics, and treatment with (neo)adjuvant chemotherapy (Table 1).

We identified six variants on chromosome 15 with genome-wide significant associations within the subgroup of patients diagnosed with an ER+ or PR+, HER2− tumor (Table 1). We identified two independent variants, namely rs8030394 and rs112813972, both situated in an intronic region of *THSD4* (Additional file 2: Supplementary Figure S5). For the most significant variant, rs8030394, the T allele was associated with increased risk of breast cancer death (HR [95% CI]: 2.47 [1.81, 3.37], P = 1.1E−08, BFDP = 0.42). For the second variant, rs112813972, the C allele was associated with decreased risk of death (HR [95% CI] 0.40 [0.28,0.55], P = 4.0E−08, BFDP = 0.70). These associations were not genome-wide significant after adjusting for age at diagnosis, additional tumor characteristics, and treatment with (neo)adjuvant chemotherapy, and the corresponding BFDPs increased to 0.72 and 0.90, respectively (Table 1).

We observed genome-wide significant associations from the 15-year breast cancer-specific analyses in the subgroups of patients with an ER+ or PR+ and HER2− tumor who did and did not receive chemotherapy. Three correlated variants on chromosome 2 were identified within the subgroup of patients who received chemotherapy. The most significant variant, rs62192052, is located in an intronic region of *DNER* (Additional file 2: Supplementary Figure S5) and was associated with decreased risk of death (HR [95% CI] 0.15 [0.08, 0.28], P = 2.6E−09, per T allele; Table 1). Although the result remained genome-wide significant after adjusting for age at diagnosis and additional tumor characteristics, the BFDP from both the unadjusted and adjusted analysis was ≥ 0.99 (Table 1; Supplementary Table S8), indicating that this association is almost certainly a false positive. Variant rs56248395, located on chromosome 11 in an intron of *NAV2* (Additional file 2: Supplementary Figure S5), was associated with breast cancer death in the subgroup of patients with an ER+ or PR+ and HER2− tumor who did not receive chemotherapy (HR [95% CI] 2.33 [1.72,3.15], P = 4.8E−08, per T allele; Table 1). This result had BFDP ≥ 0.59 and was only based on the OncoArray data, since on iCOGS the variants did not satisfy the inclusion criteria for genotypes (iCOGS imputation *r*^2^ = 0.66; Additional file 2: Supplementary Table S7). The corresponding estimates in the iCOGS data (HR [95% CI] 1.07 [0.80,1.41], P = 6.6E−01; Additional file 2: Supplementary Table S7) and from the meta-analysis (HR [95% CI] 1.53 [1.25,1.89], P = 5.2E−05; Table 2) were not genome-wide significant and not noteworthy (meta-analysis BFDP≥0.97; Table 2; Supplementary Table S9).

We observed three additional single SNP noteworthy associations from the 15-year breast cancer-specific survival analyses. The intergenic variant rs4679741 on chromosome 3 was associated with breast cancer death in the subgroup of patients with an ER+ tumor treated with endocrine therapy (HR [95% CI] 1.18 [1.11, 1.26], P = 1.6E−07, BFDP = 0.09, per G allele). This result became genome-wide significant after adjusting for age at diagnosis, tumor characteristics, and treatment with chemotherapy (HR [95% CI] 1.20 [1.13, 1.28], P = 1.1E−08, BFDP = 0.01). The BFDP of this association remained < 0.15 when considering 10^−5^ as prior probability of true association (Additional file 3: Supplementary Table S8). PanCanQTL did not show any cis-eQTLs, trans-eQTLs nor survival eQTLs for this variant. Variant rs1106333 on chromosome 3, whose nearest gene is *GRIP2*, was associated with risk of dying of breast cancer in the subgroup of patients with an ER− tumor who received chemotherapy (HR [95% CI] 1.68 [1.39,2.03], P = 5.6E−08, BFDP = 0.12 per A allele). This result also became genome-wide significant after adjusting for additional prognostic factors (HR [95% CI] 1.70 [1.41,2.05], P = 4.4E−08, BFDP = 0.11). PanCanGTL revealed the presence of a cis-eQTL linking variant rs1106333 with *GRIP2* expression in prostate adenocarcinoma but not in breast cancer. There was no evidence of association of *GRIP2* expression levels with distant metastasis-free survival within ER− breast cancer patients treated with chemotherapy based on KMPlotter data (Additional file 2: Supplementary Figure S11). The last association of interest was observed in the subgroup of patients who received anthracyclines with intergenic variant rs34072391 on chromosome 7, but was not noteworthy after adjustment for additional prognostic factors (Table 1).

In the 5-year survival analyses focused on the treatment subgroups, we observed two genome-wide significant associations within the subgroup of patients diagnosed with an ER− tumor who received chemotherapy. The most significant variant was rs78754389, located on chromosome 4 in an intronic region of gene *ARAP2* (Additional file 2: Supplementary Figure S5; HR [95% CI] 1.79 [1.46,2.20], P = 1.7E−08, BFDP = 0.07 per A allele). This result remained both genome-wide significant and noteworthy after adjusting for age at diagnosis and additional tumor characteristics (HR [95% CI] 1.67 [1.39,2.00], P = 4.1E−08, BFDP = 0.09; Table 1). However, PanCanQTL did not show any cis-eQTLs, trans-eQTLs nor survival eQTLs for rs78754389 and there was no evidence of association of *ARAP2* expression levels with distant metastasis-free survival within ER− breast cancer patients treated with chemotherapy based on KMPlotter data (Additional file 2: Supplementary Figure S12). The second genome-wide significant variant was rs117685664, located on chromosome 8 (HR [95% CI] 0.26 [0.16,0.42], P = 4.6E−08, per T allele). This association was not genome-wide significant after accounting for the age at diagnosis and additional tumor characteristics (Table 1), and the corresponding BFDPs from unadjusted and adjusted analysis were 1.00 and 0.99, respectively, indicating a false positive finding.

We also observed one additional noteworthy but not genome-wide significant association in the 5-year breast cancer-specific survival analyses in the subgroup of patients who received Tamoxifen with variant rs72775397, situated in the 3′ untranslated region of *MCTP1* (HR [95% CI] 1.36 [1.21,1.53], P = 1.8E−07, BFDP = 0.11, per C allele). The association was attenuated after adjustment for additional prognostic factors (HR [95% CI] 1.11 [1.04,1.20], P = 3.8E−03, BFDP = 1.00).

We did not identify any genome-wide significant or noteworthy association in any of the remaining seven subgroups investigated (Figs. 1 and 2). In addition, none of the above reported associations have a BFDP < 0.15 when considering 10^−6^ as prior probability of true association (Additional file 3: Supplementary Tables S8). Moreover, none of the subgroup-specific genome-wide significant associations detected by previous studies using a smaller version (both in terms of number of cases and of length of follow-up) of the iCOGS and/or OncoArray BCAC datasets were replicated at P < 0.001 (Additional file 3: Supplementary Table S12).

## Discussion

We investigated the association of over 10 million common germline genetic variants with breast cancer-specific survival within 15 patient subgroups based on prognostic factors representative of tumor biology or related to the type of systemic treatment. Our hypothesis was that focusing on more homogeneous subgroups of breast cancer patients might reveal otherwise undetected associations. Besides type of systemic treatment, the definition of the subgroups was based on current clinically used biological characteristics for tumor subtyping and treatment decisions: patient’s age, tumor histological grade, ER, PR, and HER2 status ( [31]; Supplementary table S2). We did not have gene expression or copy number aberration data available to classify tumors on the basis of specific biological processes [44, 45]; however, relevant survival differences have been reported among the four subtypes based on ER, PR, and HER2 status [27, 46, 47].

A concern about performing GWAS on several subgroups of patients is the increased proportion of false discoveries, also known as type I errors. For this reason and to overcome additional limitations of the association p values [35–37], we made use of the BFDP approach and only considered as robust candidates those associations with BFDP < 0.15 at a prior probability of 10^−4^.

We found evidence of four loci potentially associated with breast cancer survival: one in the subgroup of patients diagnosed with a grade 3 tumor, one in the subgroup of patients with an ER+ tumor and treated with endocrine therapy, and two in the subgroup of patients with an ER− tumor and treated with chemotherapy.

The most significant variant identified in the subgroup of grade 3 tumors, rs5934618, is situated in intron 1 of *TBL1X*, a gene which encodes the Transducin (beta)-like 1X-linked protein. Both *TBL1X* and the closely related gene *TBLR1* have been implicated in the activation of the Wnt/beta-catenin signaling pathway, which has been reported to be overactivated in the progression and proliferation of several tumors, including breast tumors, where it has been linked with reduced overall survival [48–50]. Rs5934618 and the other four correlated variants identified in our genome-wide analysis overlap with chromatin features H3K4me3 and H3K27ac in normal breast; these histone marks are generally characteristics of gene promoters and/or enhancers and might indicate that one or more of these variants act through modulating expression of *TBL1X*. There was no direct evidence that any of these variants are expression single-nucleotide polymorphisms (eSNPs) for *TBL1X*, but this might reflect the tissues examined or that the variants only regulate the gene in a specific context.

The remaining three variants potentially associated with breast cancer survival were as follows: rs4679741, identified in the subgroup of patients diagnosed with an ER+ tumor and treated with endocrine therapy; rs1106333 and rs78754389, identified in the subgroup of patients diagnosed with an ER− tumor and treated with chemotherapy. For variant rs4679741, it is unclear which the potential target genes might be, while there was no evidence linking the other two variants to the expression of the closest genes in breast cancer nor evidence of association between those genes and survival within the specific subgroups of breast cancer patients. Nevertheless, there are several mechanisms through which the four identified variants could affect survival. For example, they could act through regulation of an unannotated long noncoding RNA [51, 52] or microRNA [53, 54]. Further functional studies including epigenetic mechanisms are needed in order to gain more insights about the detected associations and to ascertain the potential underlying biological mechanisms.

We did not find strong evidence of germline variants associated with breast cancer-specific survival in any of the other subgroups of patients investigated. In addition, we did not replicate any of the subgroup-specific associations identified by previous studies. One of these associations, with variant rs4458204, was previously detected in the subgroup of patients with an ER− tumor who received chemotherapy [16]. The estimated HR (95% CI) was 1.81 (1.49–2.19) with association P = 1.9E−09. In our analysis of the same subgroup, we obtained a much lower HR estimate and the association was no longer statistically significant (HR (95% CI) 1.14 (0.99, 1.32), P = 6.0E−02), suggesting that the previous result was a false positive. Even though there is some overlap in terms of patients between the previous study and our current study, the latter is based on a substantially larger number of breast cancer patients and it includes more complete follow-up data.

A major strength of our study is the sample size, which was the largest to date and provided reasonable power to detect associations with breast cancer-specific survival within specific subgroups of patients. On the other hand, our study is subject to several limitations that are intrinsic to large consortium studies: these include variation in study design, time periods of diagnosis, and duration of follow-up, all of which can contribute to within subgroup heterogeneity. Some broad treatment-related subgroups, namely ER+ treated with any endocrine therapy and ER− treated with any chemotherapy, may include different treatments due to the wide period of diagnosis included in our study. On the other hand, the majority of patients were diagnosed between 2000 and 2009 (69.9% and 64.5% for ER+ treated with any endocrine therapy and ER− treated with any chemotherapy, respectively). If any impact on the results, the variation in treatment over time might have hampered the detection of associations between variants and survival in these subgroups. Several studies did not report the cause of death for all patients. Out of 14,606 deaths observed within the first 15 years after diagnosis, 7531 (51.6%) were due to breast cancer. Of the remaining 7075 deaths, 4905 (33.6%) were due to causes other than breast cancer, and for 2170 deaths (14.8%) it was unknown whether they were due to breast cancer or to other causes. This will have led to a loss of power, given that most of the deaths of unknown cause are likely to have been due to breast cancer. A related weakness of the study is its dependence on accuracy of cause of death certification and on coding practices of underlying cause of death in different countries. However, despite potential inaccuracies in cause of death, we considered it more valid to focus on deaths reported as due to breast cancer than by considering all deaths together, which would include those due to other causes. An additional limitation of the study is that in most subgroups we had very limited power to detect highly significant associations, particularly for small to moderate effect sizes (HRs 1.05–1.30), even for variants of relatively high minor allele frequency (MAF = 0.20). Therefore, we may have missed variants with low to moderate associations with survival.

## Conclusions

In conclusion, we found evidence of four loci associated with breast cancer-specific survival within specific patient subgroups. The variants identified appear to be independent of known additional prognostic factors, as shown in the results of the adjusted analyses based on imputed clinic-pathological variables, and could, after proper validation, improve prognostic estimates and potentially help in better stratifying patients in treatment subgroups. However, the power for many subgroups is limited due to the low number of events. Even so, given the lack of evidence of strong associations in many of the patient subgroups investigated, and the fact that previously reported variants were not confirmed, our results suggest that the impact of common germline genetic variant on breast cancer-specific survival might be limited.

## Supplementary Information

**Additional file 1: Supplementary Table S1**, **Supplementary Table S2**, **Supplementary Table S3**, and **Supplementary Table S4**. **Supplementary Table S1.** Shows an overview of the Breast Cancer Association Consortium studies included in the analyses. **Supplementary Table S2.** Includes the rationale and references to the literature supporting the choice of each subgroup for inclusion in a genome-wide association study on breast cancer-specific survival. **Supplementary Table S3.** Shows the characteristics of the breast cancer patients included in the 15-year breast cancer-specific survival analyses, overall and by subgroup. **Supplementary Table S4.** Shows the characteristics of the breast cancer patients included in the 5-year breast cancer-specific survival analyses for subgroups defined based on type of systemic treatment received.

**Additional file 2: Supplementary Methods**, **Supplementary Table S5**, **Supplementary Table S6**, **Supplementary Table S7**, **Supplementary Figure S1**, **Supplementary Figure S2**, **Supplementary Figure S3**, **Supplementary Figure S4**, **Supplementary Figure S5**, **Supplementary Figure S6**, **Supplementary Figure S7**, **Supplementary Figure S8**, **Supplementary Figure S9**, **Supplementary Figure S10**, **Supplementary Figure S11**, **Supplementary Figure S12**. The Supplementary methods section includes details about: multiple imputation of missing data; power calculations. **Supplementary Table S5.** Shows an overview of number of breast cancer patients, breast cancer deaths and follow-up information by subgroup and endpoint. **Supplementary Table S6.** Shows a list of imputed variables with corresponding percentage of missing values, imputation method and processing. **Supplementary Table S7.** Shows an overview of the GWAS significant associations (P < 5 E-08) and noteworthy (BFDP< 0.15) associations from the unadjusted 15-year and 5-year breast cancer-specific survival analyses by subgroup. **Supplementary Figure S1.** Shows the Q-Q plots of the meta-analysis results of all variants for 15-year breast cancer-specific survival. **Supplementary Figure S2.** Shows the Q-Q plots of the meta-analysis results of all variants for 5-year breast cancer-specific survival. **Supplementary Figure S3.** Shows the Q-Q plots of the meta-analysis results for 15-year breast cancer-specific survival and the corresponding genome inflation factors by minor allele frequency. **Supplementary Figure S4.** Shows the Q-Q plots of the meta-analysis results for 5-year breast cancer-specific survival and the corresponding genome inflation factors by minor allele frequency. **Supplementary Figure S5.** Shows the regional plots of genome-wide significant (P < 5E-08) independent associated variants from the 15-year and 5-year genome-wide breast cancer-specific survival analyses. **Supplementary Figure S6.** Shows the regional plots of noteworthy (BFDP< 0.15), non-genome-wide significant (P > 5E-08) variants from the 15-year and 5-year genome-wide breast cancer-specific survival analyses. **Supplementary Figure S7** shows the unadjusted association of variant rs5934618 with 15-year breast cancer-specific survival by tumor grade and in all breast cancer patients. **Supplementary Figure S8.** Shows the functional annotation and position of variants rs5934618, rs4830644, rs3810742, rs4830642, and rs72611496 relative to *TBL1X*. **Supplementary Figure S9.** Shows Kaplan-Meier distant metastasis-free survival plots (based on KMPlotter data) for high versus low expression level of gene *TBL1X* by tumor grade. **Supplementary Figure S10.** Shows the association of genetic variants with *TBL1X* expression, based on GTEx v8 data on samples of normal breast tissue from 396 individuals (male and female). **Supplementary Figure S11.** Shows a Kaplan-Meier distant metastasis-free survival plot for high versus low expression level of gene *GRIP2*, restricted to patients with an ER- tumor who received chemotherapy (based on KMPlotter data). **Supplementary Figure S12.** Shows a Kaplan-Meier distant metastasis-free survival plot for high versus low expression level of gene *ARAP2*, restricted to patients with an ER- tumor who received chemotherapy (based on KMPlotter data).

**Additional file 3: Supplementary Table S8**, **Supplementary Table S9**, **Supplementary Table S10**, **Supplementary Table S11**, and **Supplementary Table S12**. **Supplementary Table S8.** Shows the BFDPs under two more restrictive prior probabilities of true association (10^-5^ and 10^-6^) for the results presented in Table 1. **Supplementary Table S9.** Shows the BFDPs under two more restrictive prior probabilities of true association (10^-5^ and 10^-6^) for the results presented in Table 2. **Supplementary Table S10.** Shows power calculation by subgroup, at the two-sided 5E-08 level for varying genotype hazard ratio (GHR) and minor allele frequency (MAF), based on number of cases and event rate from the 15-year breast cancer-specific analyses. **Supplementary Table S11.** Shows power calculation by subgroup, at the two-sided 5E-08 level for varying genotype hazard ratio (GHR) and minor allele frequency (MAF), based on number of cases and event rate from the 5-year breast cancer-specific analyses. **Supplementary Table S12.** Shows the subgroup-specific associations detected by previous studies and corresponding estimates from the current study.

## Data Availability

All estimates from the genome-wide survival analyses are available through the BCAC website: http://bcac.ccge.medschl.cam.ac.uk. The datasets analyzed during the current study are not publicly available due to protection of participant privacy and confidentiality, and ownership of the contributing institutions, but may be made available in an anonymized form via the corresponding author on reasonable request and after approval of the involved institutions. To receive access to the data, a concept form must be submitted, which will then be reviewed by the BCAC Data Access Coordination Committee (DACC); see http://bcac.ccge.medschl.cam.ac.uk/bcacdata/.

## Abbreviations

BCAC: Breast Cancer Association Consortium
BFDP: Bayesian false discovery probability
CI: Confidence interval
CMF: Cyclophosphamide Methotrexate Fluorouracil
EBCTCG: Early Breast Cancer Trialists’ Collaborative Group
ER: Estrogen receptor
eQTL: Expression quantitative trait locus
eSNP: Expression single-nucleotide polymorphism
FUMA: Functional mapping and annotation
GWAS: Genome-wide association study
HER2: Human epidermal growth factor receptor 2
HR: Hazard ratio or Hormone receptor
HRC: Haplotype Reference Consortium
MAF: Minor allele frequency
MICE: Multiple imputation by chained equations
PR: Progesterone receptor
SNV: Single-nucleotide variant
