# Supplementary material for: Association of germline genetic variants with breast cancer-specific survival in patient subgroups defined by clinic-pathological variables related to tumor biology and type of systemic treatment
Source: Breast Cancer Res. 2021 Aug 18;23:86. doi: 10.1186/s13058-021-01450-7 (PMC8371820; doi:10.1186/s13058-021-01450-7)
Supplement: Supplementary file 2 — Additional file 2: Supplementary Methods, Supplementary Table S5, Supplementary Table S6, Supplementary Table S7, Supplementary Figure S1, Supplementary Figure S2, Supplementary Figure S3, Supplementary Figure S4, Supplementary Figure S5, Supplementary Figure S6, Supplementary Figure S7, Supplementary Figure S8, Supplementary Figure S9, Supplementary Figure S10, Supplementary Figure S11, Supplementary Figure S12. The Supplementary methods section includes details about: multiple imputation of missing data; power calculations. Supplementary Table S5. Shows an overview of number of breast cancer patients, breast cancer deaths and follow-up information by subgroup and endpoint. Supplementary Table S6. Shows a list of imputed variables with corresponding percentage of missing values, imputation method and processing. Supplementary Table S7. Shows an overview of the GWAS significant associations (P < 5 E-08) and noteworthy (BFDP< 0.15) associations from the unadjusted 15-year and 5-year breast cancer-specific survival analyses by subgroup. Supplementary Figure S1. Shows the Q-Q plots of the meta-analysis results of all variants for 15-year breast cancer-specific survival. Supplementary Figure S2. Shows the Q-Q plots of the meta-analysis results of all variants for 5-year breast cancer-specific survival. Supplementary Figure S3. Shows the Q-Q plots of the meta-analysis results for 15-year breast cancer-specific survival and the corresponding genome inflation factors by minor allele frequency. Supplementary Figure S4. Shows the Q-Q plots of the meta-analysis results for 5-year breast cancer-specific survival and the corresponding genome inflation factors by minor allele frequency. Supplementary Figure S5. Shows the regional plots of genome-wide significant (P < 5E-08) independent associated variants from the 15-year and 5-year genome-wide breast cancer-specific survival analyses. Supplementary Figure S6. Shows the regional plots of noteworthy (BFDP< 0.15), non-genome-wide [file 13058_2021_1450_MOESM2_ESM.docx]

**Supplementary methods**

**Multiple imputation of missing data**

Multiple imputation was performed after applying the exclusion criteria for individual subjects and studies. Ten imputed datasets were generated using the R package MICE (version 3.2.0) through 30 iterations of the multivariate imputation by chained equations (MICE) algorithm.

The list of imputed variables, with corresponding percentage of missing values, specific imputation method, and additional information about pre-processing of the data and imputation post-processing can be found in Supplementary Table S3. Pre-processing refers to adjustments in the data made before imputation. Post-processing refers to the procedure implemented in the MICE R package, which allows modification of imputed values directly after imputation, based on specified conditions. Variables have been imputed in ascending order of the number of missing values.

For each imputed variable, predictors to be included in the corresponding imputation model were selected among the other variables listed in Supplementary Table S3 if their correlation coefficient with the variable to impute was larger than 0.125 and their proportion of observed values among the cases with missing data on the variable to impute was at least 0.200. Tumor size category was added as predictor in the imputation model of tumor size in mm, and neo-adjuvant chemotherapy status (yes vs no) was added as predictor in the imputation models of neo-adjuvant anthracyclines status, neo-adjuvant taxanes status and neo-adjuvant CMF-like chemotherapy status. The variable “study” was included in all imputation models, in order to preserve the heterogeneity among studies.

In order to improve imputation, the Nelson-Aalen estimator of the baseline cumulative hazard and the event indicator of breast cancer-specific survival, were included in all imputation models [1].

Estimates from the analyses across different imputed datasets were combined via the Rubin’s rule [2, 3]. For more details related to the multiple imputation approach used, we refer to the book of Stef van Buuren [4].

**Power calculations**

Power calculations were performed, by subgroup, using the R package survSNP [5]. Power was estimated at varying genotype hazard ratios (1.05-1.80) and minor allele frequencies (0.01-0.5) for a type 1 error equal to 5E-08 and considering an additive model for the genetic effect. Sample sizes and event rates were the ones observed in each of the subgroups investigated (Supplementary Table S2, S5 and S6).

1. White, I.R. and P. Royston, *Imputing missing covariate values for the Cox model.* 2009. **28**(15): p. 1982-1998.

2. Rubin, D.B., *Multiple Imputation for Nonresponse in Surveys*. Wiley Series in Probability and Statistics, ed. I. John Wiley & Sons. 1987, New York: John Wiley & Sons, Inc.

3. Barnard, J. and D.B. Rubin, *Small-Sample Degrees of Freedom with Multiple Imputation.* Biometrika, 1999. **86**(4): p. 948-955.

4. Buuren, S.v., *Flexible imputation of missing data*. Second Edition ed. Interdisciplinary Statistics Series. 2018: Chapman & Hall/CRC.

5. Owzar, K., et al., *Power and sample size calculations for SNP association studies with censored time-to-event outcomes.* Genetic epidemiology, 2012. **36**(6): p. 538-548.

Supplementary Table S5. Overview of number of breast cancer patients (cases), breast cancer deaths (events) and follow up information (person-years) by subgroup and endpoint.

| **Subgroup** | **iCOGS** | | **OncoArray** | | **Total** | |
| --- | --- | --- | --- | --- | --- | --- |
|  | **Events/Cases** | **Person-years** | **Events/Cases** | **Person-years** | **Events/Cases** | **Person-years** |
| All patients |  |  |  |  |  |  |
| 15-year | 3151 / 31377 | 241989 | 4380 / 60309 | 412843 | 7531 / 91686 | 654832 |
| Age ≤ 40 at diagnosis |  |  |  |  |  |  |
| 15-year | 412 / 2452 | 17877 | 553 / 4829 | 33870 | 965 / 7281 | 51747 |
| Grade 3 tumors |  |  |  |  |  |  |
| 15-year | 1144 / 7821 | 56175 | 1757 / 14916 | 93060 | 2901 / 22737 | 149235 |
| ER + treated with ET |  |  |  |  |  |  |
| 15-year | 1256 / 14128 | 120811 | 1042 / 16009 | 100201 | 2298 / 30137 | 221012 |
| 5-year | 329 / 12202 | 43017 | 444 / 15138 | 56090 | 773 / 27340 | 99107 |
| ER- treated with chemotherapy |  |  |  |  |  |  |
| 15-year | 405 / 2663 | 18886 | 502 / 4189 | 25436 | 907 / 6852 | 44322 |
| 5-year | 266 / 2404 | 7850 | 372 / 4007 | 14485 | 638 / 6411 | 22335 |
| ER+ or PR+ and HER2- |  |  |  |  |  |  |
| 15-year | 887 / 10997 | 85766 | 1254 / 24553 | 143197 | 2141 / 35550 | 228963 |
| ER+ or PR+ and HER2- treated with CT |  |  |  |  |  |  |
| 15-year | 399/3057 | 24150 | 490/7337 | 36030 | 1198/10394 | 60180 |
| 5-year | 118/2977 | 11435 | 246/7296 | 25336 | 364/10273 | 36771 |
| ER+ or PR+ and HER2- not treated with CT |  |  |  |  |  |  |
| 15-year | 307/5003 | 43991 | 150/5272 | 26591 | 457/10275 | 70582 |
| 5-year | 93/4935 | 19168 | 82/5221 | 18119 | 175/10156 | 37287 |
| ER+ or PR+ and HER2+ |  |  |  |  |  |  |
| 15-years | 149 / 1443 | 9977 | 371 / 4124 | 24546 | 520 / 5567 | 34523 |
| ER- and PR- and HER2+ |  |  |  |  |  |  |
| 15-year | 107 / 737 | 4089 | 207 / 1561 | 8962 | 314 / 2298 | 13051 |
| ER- and PR- and HER2- |  |  |  |  |  |  |
| 15-year | 243 / 1854 | 11777 | 434 / 3777 | 20836 | 677 / 5631 | 32613 |
| Tamoxifen |  |  |  |  |  |  |
| 15-year | 987 / 9573 | 87475 | 790 / 7754 | 54420 | 1777 / 17327 | 141895 |
| 5-year | 305 / 8977 | 33085 | 346 / 7302 | 30021 | 651 / 16279 | 63106 |
| Aromatase inhibitors |  |  |  |  |  |  |
| 15-year | 272 / 3641 | 28319 | 223 / 5400 | 20536 | 495 / 9041 | 48855 |
| 5-year | 71 / 3518 | 13857 | 126 / 5299 | 15795 | 197 / 8817 | 29652 |
| CMF-like chemotherapy |  |  |  |  |  |  |
| 15-year | 123 / 947 | 8453 | 258 / 1716 | 13354 | 381 / 2663 | 21807 |
| 5-year | 55 / 800 | 3232 | 135 / 1544 | 6380 | 190 / 2344 | 9612 |
| Taxanes |  |  |  |  |  |  |
| 15-year | 167 / 1755 | 9857 | 295 / 3790 | 17737 | 462 / 5545 | 27594 |
| 5-year | 78 / 1714 | 6231 | 212 / 3749 | 13101 | 290 / 5463 | 19332 |
| Anthracyclines |  |  |  |  |  |  |
| 15-year | 468 / 4495 | 29835 | 824 / 6688 | 40286 | 1292 / 11183 | 70121 |
| 5-year | 204 / 4029 | 14889 | 448 / 6364 | 24361 | 652 / 10393 | 39250 |

Abbreviations: *ER* estrogen receptor, *PR* progesterone receptor, *HER2* human epidermal growth factor receptor 2, *ET* Endocrine therapy, *CMF* Cyclophosphamide Methotrexate Fluorouracil.

**Supplementary Table S6**. List of imputed variables with corresponding percentage of missing values, imputation method and processing^1^

| **Variable** | **Missing data**  **percentage** | **Pre-processing performed before imputation** | **Imputation method** | **Post-processing** |
| --- | --- | --- | --- | --- |
| Year of diagnosis | 9.5 |  | Predictive mean matching |  |
| Ethnicity | 10.4 |  | Polytomous regression |  |
| Morphology group of the tumor | 11.8 |  | Polytomous regression |  |
| ER status | 15.0 |  | Logistic regression |  |
| Lymph node status | 20.3 |  | Logistic regression |  |
| Histopathological grade | 20.8 |  | Polytomous regression |  |
| PR status | 26.1 |  | Logistic regression |  |
| Number of positive lymph nodes | 26.3 |  | Predictive mean matching |  |
| Size category of the tumor | 26.8 |  | Polytomous regression |  |
| Bilaterality status | 29.4 |  | Polytomous regression |  |
| Tumor size in mm | 32.7 |  | Predictive mean matching |  |
| TNM stage | 41.9 | If missing and corresponding value of distant metastases status = 1, then set equal to 4. | Polytomous regression |  |
| Surgery | 41.9 |  | Polytomous regression |  |
| Adjuvant CT | 42.6 |  | Logistic regression |  |
| Radiation | 45.6 |  | Polytomous regression |  |
| HER2 status | 45.9 |  | Logistic regression |  |
| Adjuvant ET | 46.1 |  | Logistic regression |  |
| Neo-adjuvant CT | 50.6 |  | Logistic regression |  |
| Anthracyclines (neo-adjuvant) | 51.8 |  | Logistic regression |  |
| Taxanes (neo-adjuvant) | 51.8 |  | Logistic regression |  |
| CMF-like CT (neo-adjuvant) | 52.0 |  | Logistic regression |  |
| CMF-like CT (adjuvant) | 52.8 |  | Logistic regression |  |
| Taxanes (adjuvant) | 52.9 |  | Logistic regression |  |
| Anthracyclines (adjuvant) | 53.2 |  | Logistic regression |  |
| Distant metastases status | 55.7 | If missing and corresponding value of TNM stage = 4, then set equal to 1. | Polytomous regression |  |
| Aromatase inhibitor | 58.0 |  | Logistic regression |  |
| Tamoxifen | 58.0 |  | Logistic regression |  |
| Trastuzumab | 59.3 | If missing and corresponding value of Year of diagnosis observed and < 1998, then set equal to 0. | Logistic regression | Only for imputed values: if corresponding imputed value of Year of diagnosis<1998, then set equal to 0. |

Abbreviations: *ER* estrogen receptor, *PR* progesterone receptor, *HER2* human epidermal growth factor receptor 2, *CT* chemotherapy, *ET* endocrine therapy, *CMF* Cyclophosphamide Methotrexate Fluorouracil.

^1^ The Nelson-Aalen estimator of the baseline cumulative hazard and the event indicator of breast cancer-specific survival were included in all imputation models to improve imputation. Pre-processing refers to adjustment in the data made pre-imputation. Post-processing refers to the procedure implemented in the MICE R package, that allow to modify imputed values directly after imputation of an imputed variable (so that the adjusted values can be used in the imputation of the other variables in the same imputed dataset within the same iteration of the algorithm).

Supplementary Table S7. GWAS significant associations (P < 5×10-8) and noteworthy (BFDP<0.15) associations from the unadjusted 15-year and 5-year breast cancer-specific survival analyses by subgroup.

| **Subgroup** | **Variant** | **Chr** | **Position** | **Alleles^a^** | **iCOGS** | | | | **OncoArray** | | | | **Meta-analysis** | | | |
| --- | --- | --- | --- | --- | --- | --- | --- | --- | --- | --- | --- | --- | --- | --- | --- | --- |
|  |  |  |  |  | **HR [95% CI]** | **P value** | **AAF** | **R^2^** | **HR [95% CI]** | **P value** | **AAF** | **R^2^** | **HR [95% CI]** | **P value** | **BFDP** | **I^2^** |
| All patients | rs57714252^b^ | 4 | 30069647 | T/C | *1.03[0.94,1.13]* | *5.1E-01* | *0.14* | *0.62* | 0.84[0.79,0.89] | 4.7E-08 | 0.14 | 1.00 | *0.90[0.85,0.95]* | *4.4E-05* | *0.95* | *0.93* |
|  | rs4129285^b^ | 4 | 30065111 | T/C | *1.03[0.94,1.13]* | *5.2E-01* | *0.14* | *0.62* | 0.84[0.79,0.89] | 4.9E-08 | 0.14 | 1.00 | *0.90[0.85,0.95]* | *4.3E-05* | *0.95* | *0.93* |
| Grade 3 tumors | rs5934618^b^ | X | 9437463 | A/G | *1.16[0.99,1.39]* | *7.7E-02* | *0.12* | *0.50* | 1.39[1.24,1.56] | 1.7E-08 | 0.08 | 0.96 | *1.32[1.20,1.45]* | *1.4E-08* | *0.01* | *0.63* |
|  | rs4830644^b^ | X | 9434808 | A/G | *1.16[0.98,1.38]* | *9.7E-02* | *0.12* | *0.51* | 1.39[1.24,1.56] | 2.0E-08 | 0.08 | 0.97 | *1.32[1.20,1.45]* | *2.1E-08* | *0.01* | *0.66* |
|  | rs3810742^b, c^ | X | 9432603 | T/C | *1.15[0.97,1.39]* | *1.1E-01* | *0.13* | *0.50* | 1.38[1.24,1.55] | 2.0E-08 | 0.08 | 1.00 | *1.31[1.19,1.44]* | *2.7E-08* | *0.02* | *0.69* |
|  | rs4830642^b^ | X | 9431786 | T/C | *1.16[0.98,1.37]* | *8.9E-02* | *0.13* | *0.50* | 1.38[1.24,1.55] | 2.9E-08 | 0.08 | 0.98 | *1.31[1.19,1.44]* | *2.8E-08* | *0.02* | *0.65* |
|  | rs72611496^b^ | X | 9434264 | G/A | *1.18[0.99,1.41]* | *7.0E-02* | *0.12* | *0.49* | 1.38[1.24,1.55] | 4.3E-08 | 0.08 | 0.98 | *1.32[1.20,1.45]* | *2.3E-08* | *0.02* | *0.52* |
|  | rs66871326 | 2 | 209048052 | AAGGAG/A | 0.85[0.77,0.94] | 2.2E-03 | 0.77 | 0.86 | 0.85[0.79,0.92] | 2.6E-05 | 0.76 | 1.00 | 0.85[0.80,0.90] | 2.1E-07 | 0.11 | 0.00 |
| ER+ or PR+, and HER2- | rs8030394 | 15 | 71637241 | C/T | 2.37[1.49,3.79] | 3.0E-04 | 0.99 | 0.98 | 2.55[1.69,3.87] | 9.5E-06 | 0.99 | 0.98 | 2.47[1.81,3.37] | 1.1E-08 | 0.42 | 0.00 |
|  | rs112641969 | 15 | 71715016 | A/G | 0.45[0.29,0.69] | 2.5E-04 | 0.02 | 0.79 | 0.47[0.32,0.68] | 4.9E-05 | 0.02 | 0.83 | 0.46[0.35,0.61] | 4.6E-08 | 0.46 | 0.00 |
|  | rs16955466 | 15 | 71637757 | C/T | 0.39[0.24,0.63] | 1.3E-04 | 0.01 | 0.98 | 0.41[0.27,0.62] | 2.9E-05 | 0.02 | 0.98 | 0.40[0.29,0.55] | 1.5E-08 | 0.49 | 0.00 |
|  | rs7165279 | 15 | 71636591 | T/C | 2.27[1.43,3.61] | 5.3E-04 | 0.99 | 0.99 | 2.53[1.67,3.83] | 1.3E-05 | 0.96 | 0.98 | 2.41[1.77,3.28] | 2.7E-08 | 0.54 | 0.00 |
|  | rs111962948 | 15 | 71656213 | G/T | 0.38[0.23,0.63] | 1.2E-04 | 0.01 | 0.96 | 0.42[0.28,0.64] | 6.0E-05 | 0.02 | 0.99 | 0.41[0.29,0.56] | 3.0E-08 | 0.61 | 0.00 |
|  | rs112813972 | 15 | 71577932 | T/C | 0.41[0.25,0.67] | 4.6E-04 | 0.02 | 0.94 | 0.39[0.25,0.60] | 2.3E-05 | 0.01 | 0.98 | 0.40[0.28,0.55] | 4.0E-08 | 0.70 | 0.00 |
| ER+ or PR+, and HER2- treated with CT | rs62192052 | 2 | 230372348 | C/T | 0.20[0.08,0.51] | 6.9E-04 | 0.02 | 0.88 | 0.12[0.05,0.27] | 7.1E-07 | 0.02 | 0.89 | 0.15[0.08,0.28] | 2.6E-09 | 0.99 | 0.00 |
|  | rs74423556^c^ | 2 | 230325234 | C/G | 0.26[0.10,0.67] | 5.2E-03 | 0.02 | 0.81 | 0.10[0.04,0.24] | 3.9E-07 | 0.02 | 1.00 | 0.16[0.08,0.30] | 2.1E-08 | 0.99 | 0.55 |
|  | rs145983608 | 2 | 230296944 | A/G | 0.28[0.11,0.74] | 1.0E-02 | 0.02 | 0.78 | 0.09[0.03,0.22] | 2.5E-07 | 0.01 | 0.90 | 0.15[0.08,0.30] | 3.8E-08 | 1.00 | 0.66 |
| ER+ or PR+, and HER2- not treated with CT | rs56248395 | 11 | 20084391 | C/T | *1.07[0.80,1.41]* | *6.6E-01* | *0.12* | *0.66* | 2.33[1.72,3.15] | 4.8E-08 | 0.13 | 0.94 | *1.53[1.25,1.89]* | *5.2E-05* | *0.97* | *0.93* |
| ER+ treated with ET | rs4679741 | 3 | 155003603 | T/G | 1.24[1.13,1.36] | 4.0E-06 | 0.48 | 0.71 | 1.13[1.04,1.24] | 4.3E-03 | 0.50 | 1.00 | 1.18[1.11,1.26] | 1.6E-07 | 0.09 | 0.50 |
| ER- treated with CT | rs78754389^d^ | 4 | 35962454 | G/A | 1.56[1.12,2.16] | 8.0E-03 | 0.07 | 0.88 | 1.96[1.51,2.55] | 3.5E-07 | 0.07 | 0.90 | 1.79[1.46,2.20] | 1.7E-08 | 0.07 | 0.16 |
|  | rs1106333 | 3 | 14562127 | C/A | 1.78[1.32,2.39] | 1.3E-04 | 0.05 | 0.88 | 1.62[1.27,2.06] | 9.6E-05 | 0.06 | 0.96 | 1.68[1.39,2.03] | 5.6E-08 | 0.12 | 0.00 |
|  | rs117685664^d^ | 8 | 26989084 | C/T | 0.26[0.13,0.50] | 8.6E-05 | 0.03 | 0.85 | 0.26[0.13,0.52] | 1.4E-04 | 0.03 | 0.91 | 0.26[0.16,0.42] | 4.6E-08 | 1.00 | 0.00 |
| Tamoxifen | rs72775397^d^ | 5 | 94266932 | C/T | 1.31[1.11,1.55] | 1.8E-03 | 0.28 | 0.99 | 1.41[1.20,1.65] | 2.3E-05 | 0.29 | 1.00 | 1.36[1.21,1.53] | 1.8E-07 | 0.11 | 0.00 |
| Anthracylines | rs34072391 | 7 | 30243729 | C/CA | 1.33[1.14,1.55] | 3.2E-04 | 0.52 | 0.71 | 1.25[1.12,1.39] | 4.3E-05 | 0.52 | 0.84 | 1.27[1.17,1.39] | 6.2E-08 | 0.04 | 0.00 |

Abbreviations: *Chr* chromosome, *ER* estrogen receptor, *PR* progesterone receptor, *HER2* human epidermal growth factor receptor 2, *ET* Endocrine therapy, *CT* chemotherapy, *HR* hazard ratio, *CI* confidence interval, *AAF* alternative allele frequency, *R^2* imputation quality, *BFDP* Bayesian False Discovery Probability, *I^2^* statistic evaluating heterogeneity in the meta-analysis results.

^a^ Reference/alternative allele; ^b^ Variant with imputation r^2^ < 0.7 on iCOGS. iCOGS results and meta-analysis results are reported in *italics* for completeness; ^c^ Variant genotyped on OncoArray; ^d^ From the 5-year breast cancer-specific survival analyses.


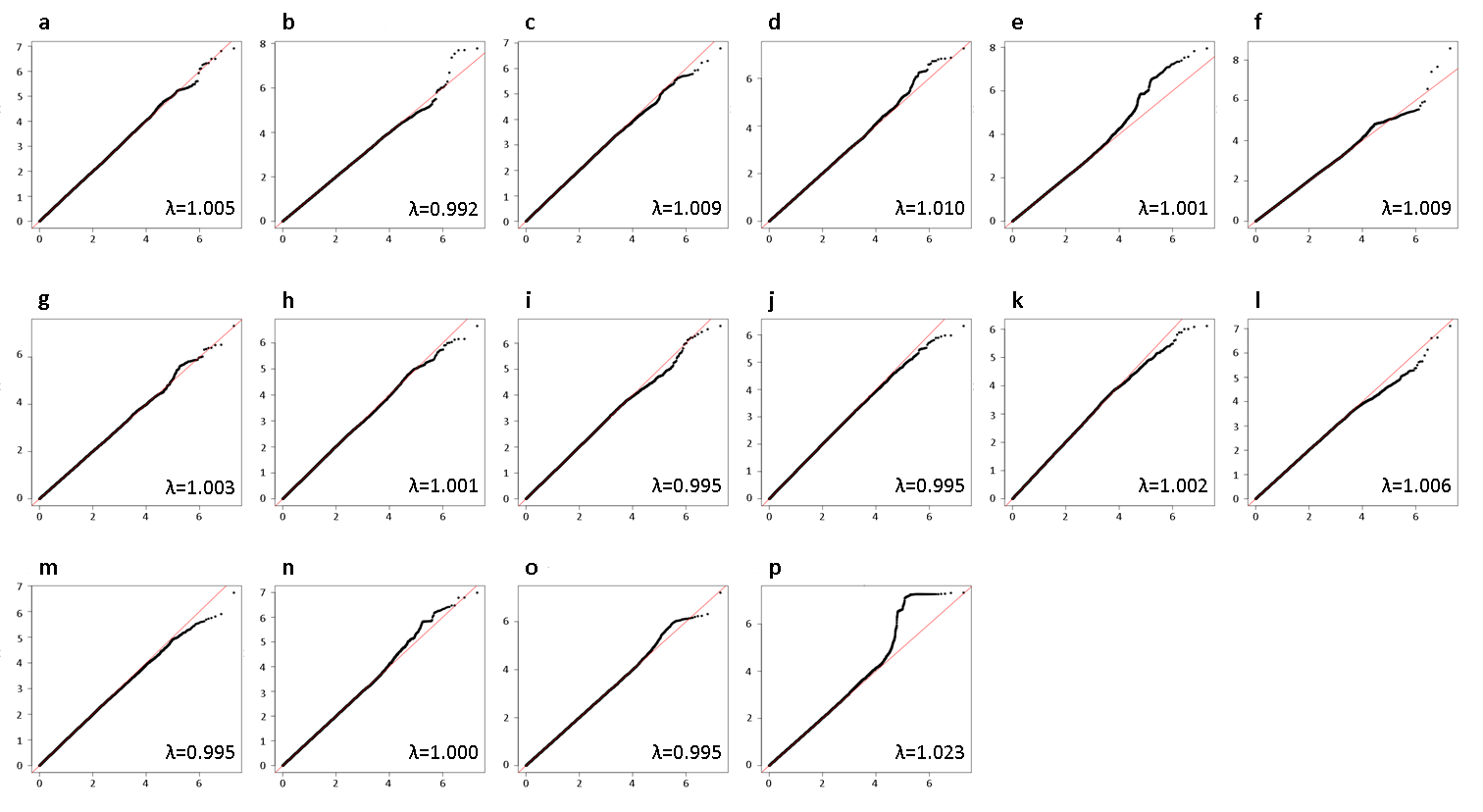


Supplementary Figure S1. Q-Q plots of the meta-analysis results of all variants for 15-year breast cancer-specific survival. The y-axis represents the observed −log_10_ p values, and the x-axis represents the expected −log_10_ p values, under the global null hypothesis of no association with the outcome.

Breast cancer patients included in the analysis in panel: a) younger than age 40 years at diagnosis; b) diagnosed with a grade 3 tumor; c) diagnosed with a ER+ tumor and treated with (any) endocrine therapy; d) diagnosed with a ER- tumor and treated with (any) chemotherapy; e) diagnosed with a ER+ or PR+, and HER2- tumor; f) diagnosed with a ER+ or PR+, and HER2- tumor treated with (any) chemotherapy; g) diagnosed with a ER+ or PR+, and HER2- tumor not treated with chemotherapy; h) diagnosed with a ER+ or PR+, and HER2+ tumor; i) diagnosed with a ER- and PR- and HER2+ tumor; j) diagnosed with a ER- and PR- and HER2- tumor; k) treated with Tamoxifen; l) treated with Aromatase inhibitor; m) treated with CMF-like chemotherapy; n) treated with Taxanes; o) treated with Anthracyclines; p) all.

Genomic inflation factor (λ).


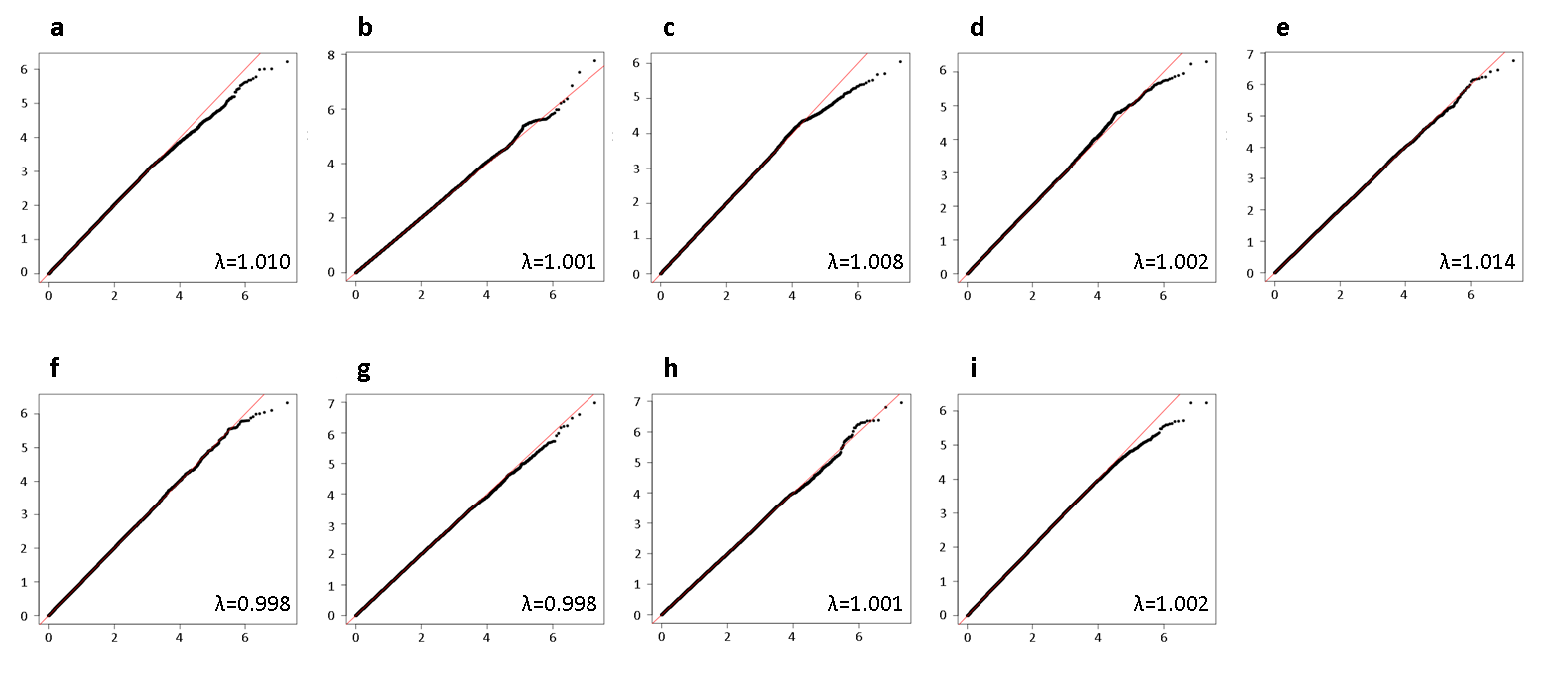


Supplementary Figure S2. Q-Q plots of the meta-analysis results of all variants for 5-year breast cancer-specific survival . The y-axis represents the observed −log_10_ p values, and the x-axis represents the expected −log_10_ p values, under the global null hypothesis of no association with the outcome.

Breast cancer patients included in the analysis in panel: a) diagnosed with a ER+ tumor and treated with (any) endocrine therapy; b) diagnosed with a ER- tumor and treated with (any) chemotherapy; c) diagnosed with a ER+ or PR+, and HER2- tumor treated with (any) chemotherapy; d) diagnosed with a ER+ or PR+, and HER2- tumor not treated with chemotherapy; e) treated with Tamoxifen; f) treated with Aromatase Inhibitor; g) treated with CMF-like chemotherapy; h) treated with Taxanes; i) treated with Anthracyclines.

Genomic inflation factor (λ).


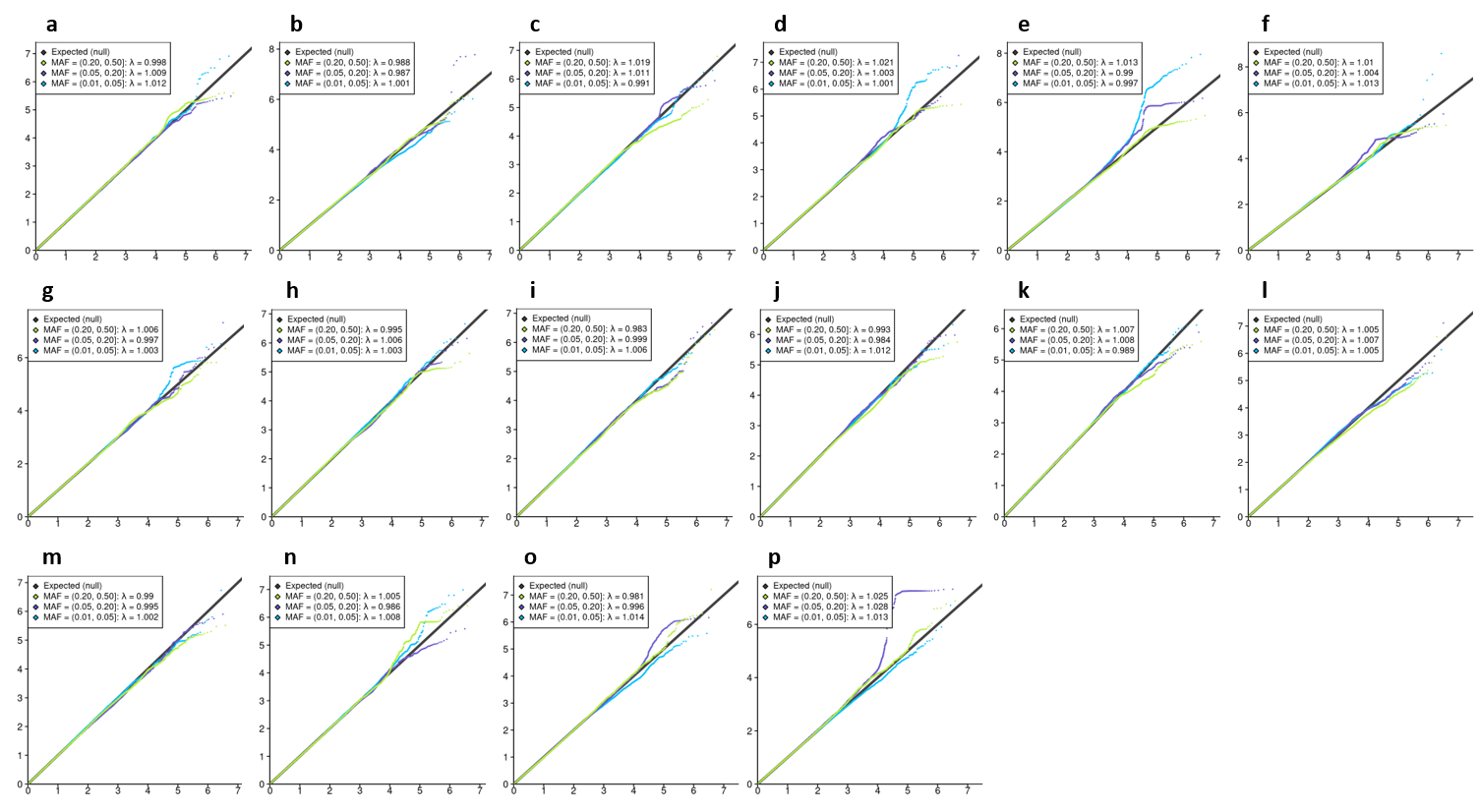


Supplementary Figure S3. Q-Q plots of the meta-analysis results of all variants for 15-year breast cancer-specific survival by minor allele frequency (MAF). The y-axis represents the observed −log_10_ p values, and the x-axis represents the expected −log_10_ p values, under the global null hypothesis of no association with the outcome.

Breast cancer patients included in the analysis in panel: a) younger than age 40 years at diagnosis; b) diagnosed with a grade 3 tumor; c) diagnosed with a ER+ tumor and treated with (any) endocrine therapy; d) diagnosed with a ER- tumor and treated with (any) chemotherapy; e) diagnosed with a ER+ or PR+, and HER2- tumor; f) diagnosed with a ER+ or PR+, and HER2- tumor treated with (any) chemotherapy; g) diagnosed with a ER+ or PR+, and HER2- tumor not treated with chemotherapy; h) diagnosed with a ER+ or PR+, and HER2+ tumor; i) diagnosed with a ER- and PR- and HER2+ tumor; j) diagnosed with a ER- and PR- and HER2- tumor; k) treated with Tamoxifen; l) treated with Aromatase inhibitor; m) treated with CMF-like chemotherapy; n) treated with Taxanes; o) treated with Anthracyclines; p) all.

λ: Genomic inflation factor by minor allele frequency.


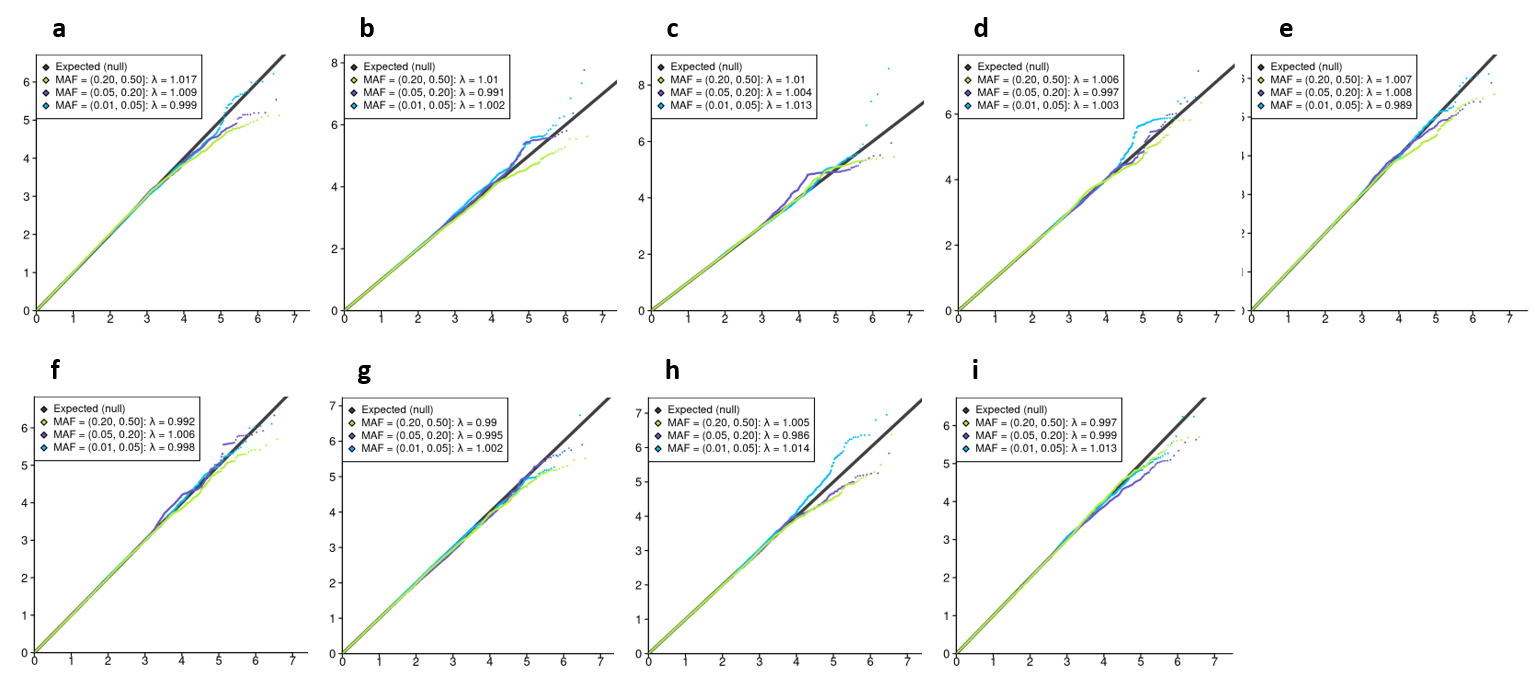


Supplementary Figure S4. Q-Q plots of the meta-analysis results of all variants for 5-year breast cancer-specific survival by minor allele frequency (MAF). The y-axis represents the observed −log_10_ p values, and the x-axis represents the expected −log_10_ p values, under the global null hypothesis of no association with the outcome.

Breast cancer patients included in the analysis in panel: a) diagnosed with a ER+ tumor and treated with (any) endocrine therapy; b) diagnosed with a ER- tumor and treated with (any) chemotherapy; c) diagnosed with a ER+ or PR+, and HER2- tumor treated with (any) chemotherapy; d) diagnosed with a ER+ or PR+, and HER2- tumor not treated with chemotherapy; e) treated with Tamoxifen; f) treated with Aromatase Inhibitor; g) treated with CMF-like chemotherapy; h) treated with Taxanes; i) treated with Anthracyclines.

λ: Genomic inflation factor by minor allele frequency.


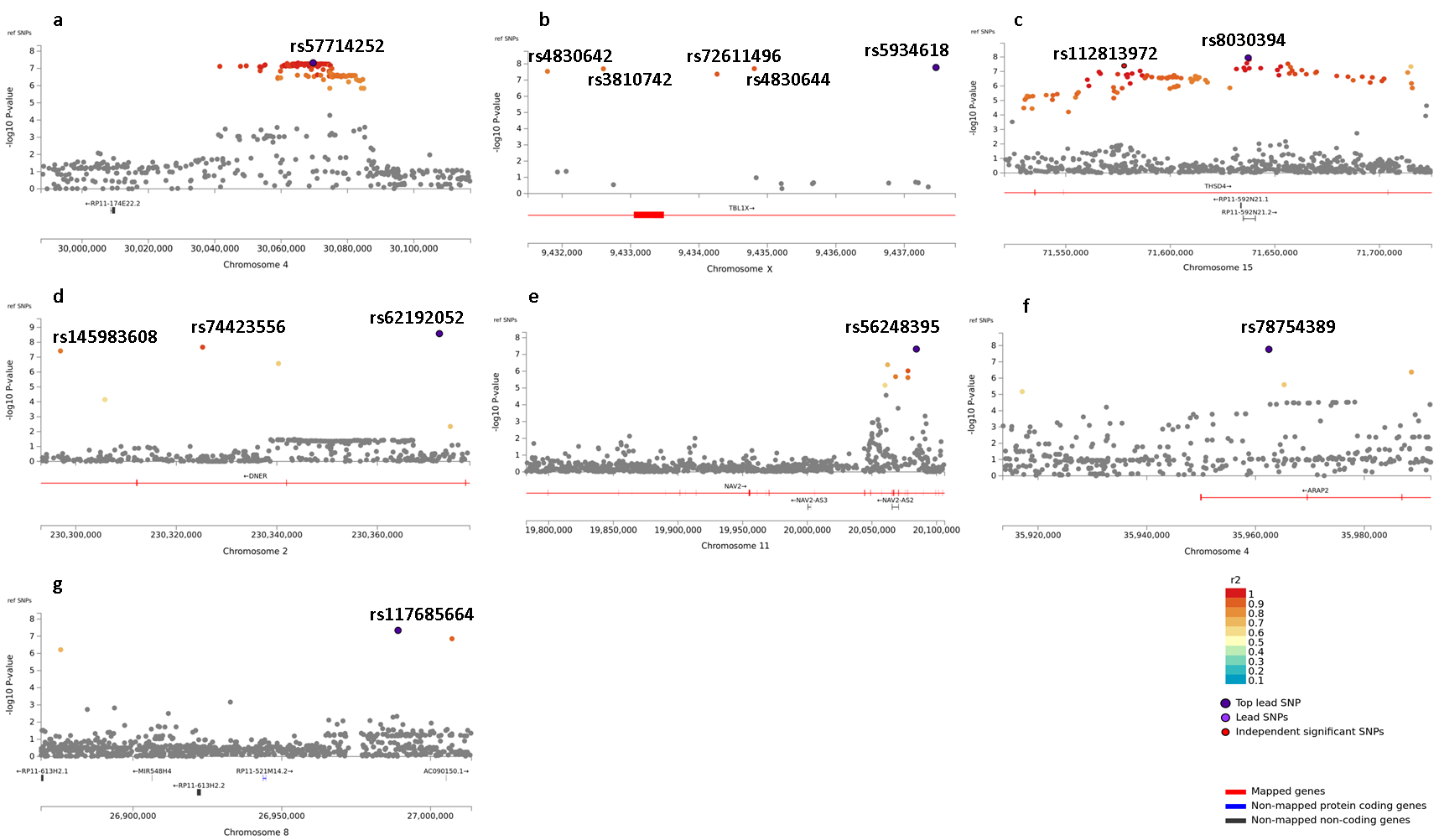


Supplementary Figure S5. Regional plots of genome-wide significant (P<5E-08) independent associated variants from:

Panel a: 15-year breast cancer-specific survival, patients diagnosed with a grade 3 tumor; b: 15-year breast cancer-specific survival, all breast cancer patients; c: 15-year breast cancer-specific survival, patients diagnosed with a ER+ or PR+, and HER2- tumor; d: 15-year breast cancer-specific survival, patients diagnosed with a ER+ or PR+, and HER2- tumor treated with (any) chemotherapy; e: 15-year breast cancer-specific survival, patients diagnosed with a ER+ or PR+, and HER2- tumor not treated with chemotherapy; f and g: 5-year breast cancer-specific survival patients diagnosed with a ER- and treated with (any) chemotherapy.

In each panel, the dark-blue dot identifies the most significant variant. In panel b, the dot circled in black and colored in red represents a second significant variant independent from the most significant one (dark-blue dot). The other variants are color-coded based on their linkage disequilibrium (r^2^) with one of the independent significant variants, from darker light blue (r^2^ < 0.2) to red (r^2^ > 0.9). Variants below the threshold of 0.1 for r^2^) are colored in grey.


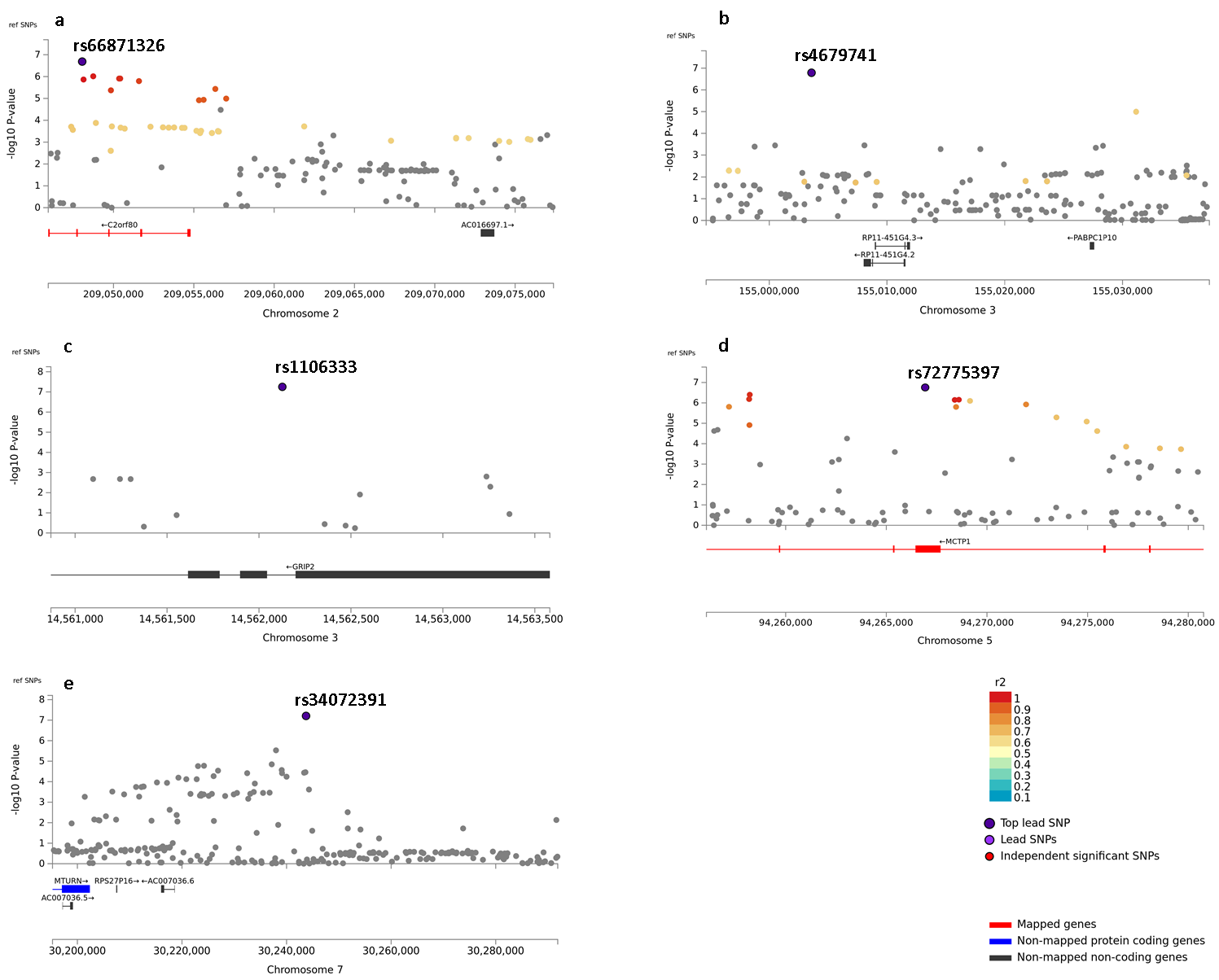


Supplementary Figure S6. Regional plots of noteworthy (BFDP<0.15), non-genome-wide significant (P>5E-08) variants from:

Panel a: 15-year breast cancer-specific survival, patients diagnosed with a grade 3 tumor; b: 15-year breast cancer-specific survival, patients diagnosed with an ER+ tumor and treated with (any) endocrine therapy; c: 15-year breast cancer-specific survival, patients diagnosed with an ER- tumor and treated with (any) chemotherapy; d: 5-year breast cancer-specific survival, patients treated with Tamoxifen; e: 15-year breast cancer-specific survival, patients treated with Anthracyclines.

In each panel, the dark-blue dot identifies the most significant variant. The other variants are color-coded based on their linkage disequilibrium (r^2^) with the most significant variant from darker light blue (r^2^ < 0.2) to red (r^2^ > 0.9). Variants below the threshold of 0.1 for r^2^) are colored in grey.


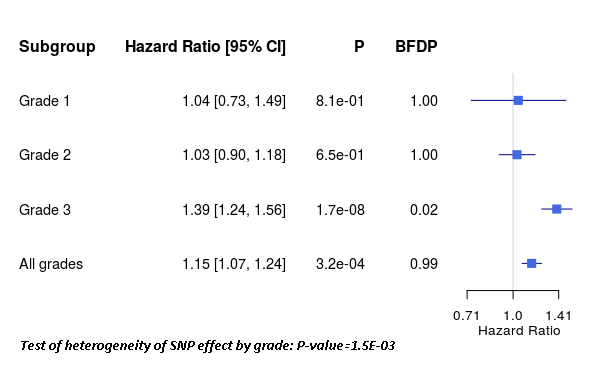


Supplementary Figure S7. Unadjusted association of variant rs5934618 with 15-year breast cancer-specific survival by tumor grade and in all breast cancer patients. The BFDP is computed assuming the prior probability of true association equal to 10-4 for all variants.


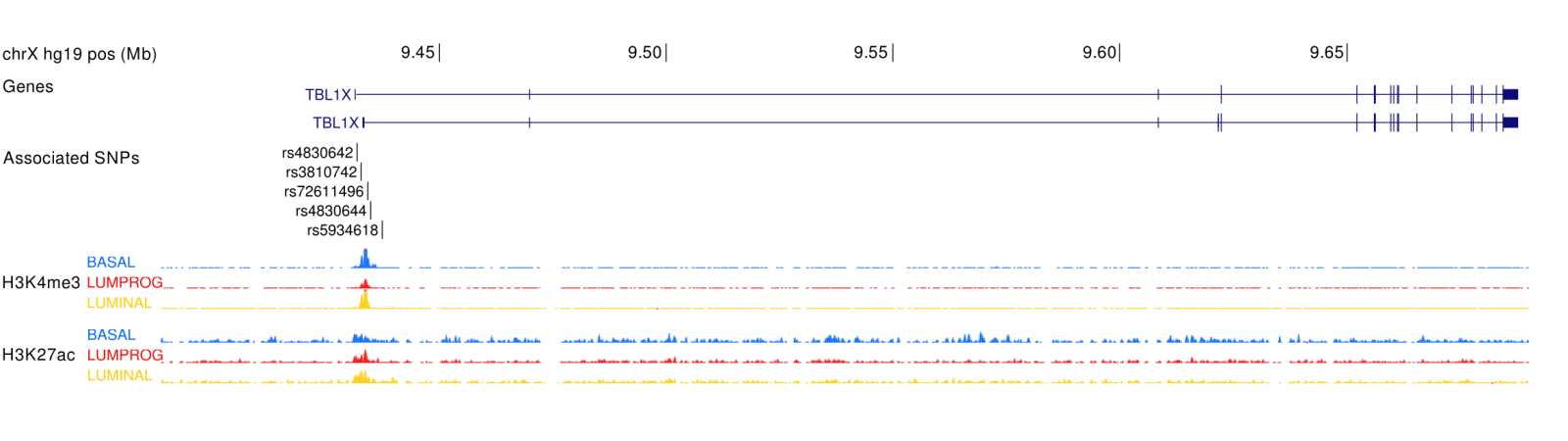


**Supplementary Figure S8**. Functional annotation and position of variants rs5934618, rs4830644, rs3810742, rs4830642, and rs72611496 relative to *TBL1X*. Chromatin histone modification ChIP-seq data from primary breast epithelial cells (basal, luminal progenitor, mature luminal), for H3K27ac (active regions) and H3K4me3 (promoters). Data from Pellacani, Cell Reports 2016.


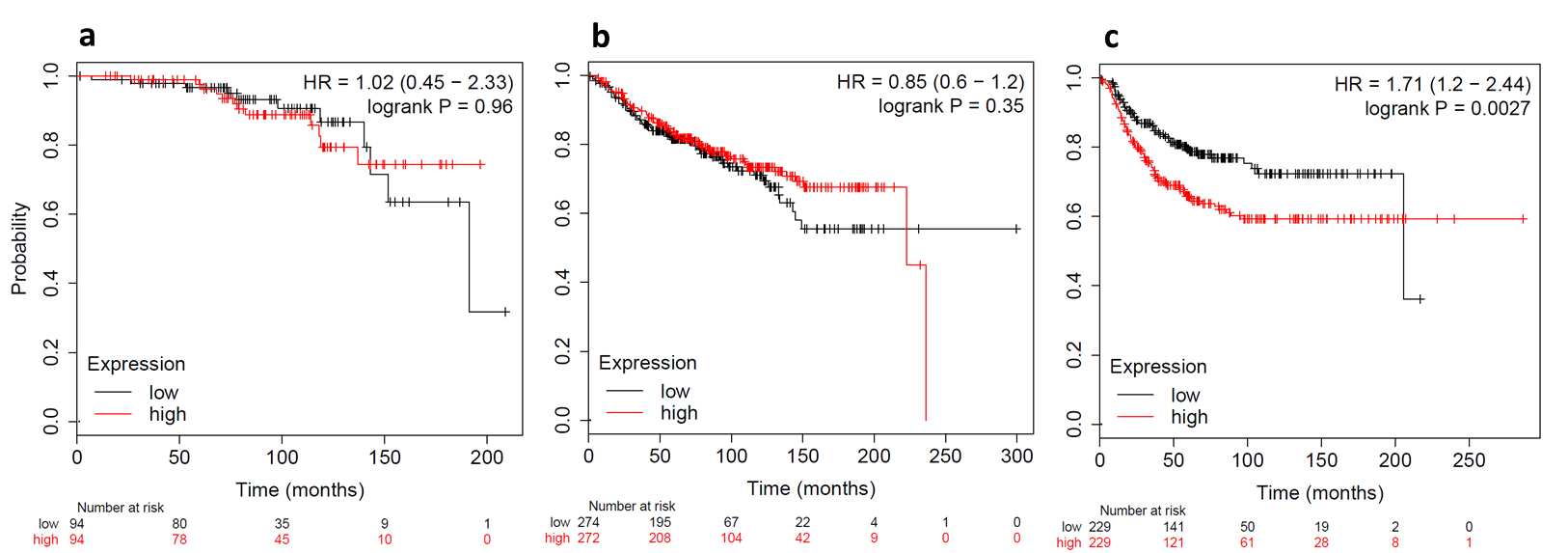


Supplementary Figure S9. Kaplan-Meier distant metastasis-free survival plots for high versus low expression level of gene *TBL1X*. The plots were generated using KMPlotter data.

Breast cancer patients included in the analysis in panel: a) 188 patients (94 with low and 94 with high *TBL1X* expression) diagnosed with a grade 1 tumor; b) 546 (274 with low and 272 with high *TBL1X* expression) patients diagnosed with a grade 2 tumor; c) 458 patients (229 with low and 229 with high *TBL1X* expression) diagnosed with a grade 3 tumor.


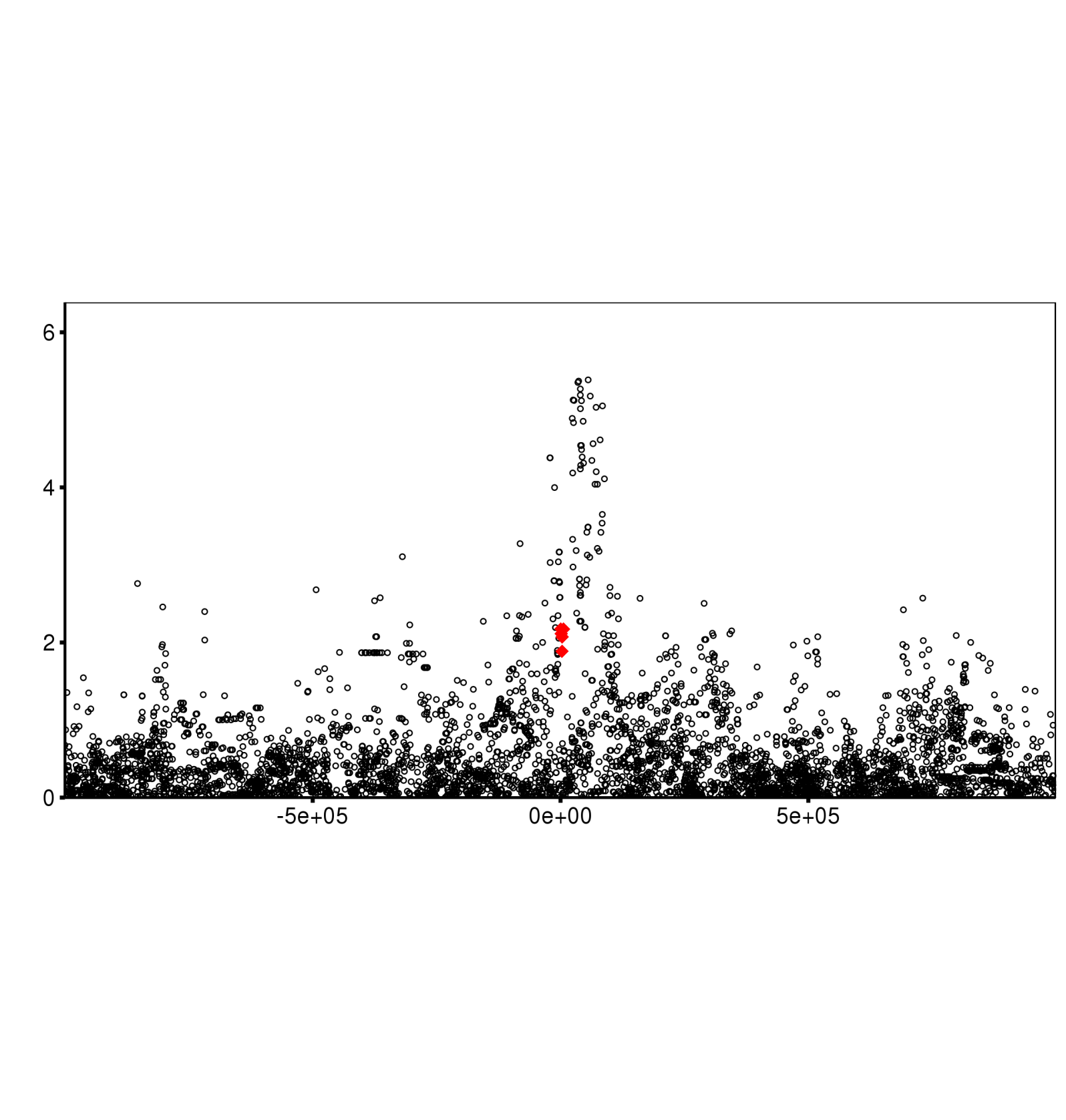


**Supplementary Figure S10.** Plot showing association of genetic variants with *TBL1X* expression, based on GTEx v8 data on samples of normal breast tissue from 396 individuals (male and female). Each point represents a variant centered around the *TBL1X* promoter. The y-axis shows the log_10_ p-values of the association of individual variants with *TBL1X* expression. The x-axis represents the distance to the transcription start site of *TBL1X*. The five variants detected in our genome-wide survival analysis restricted to patients with a grade 3 tumor are represented in red. Note that these variants are weakly associated with *TBL1X* expression and are not correlated with one or more variants at the top.

**
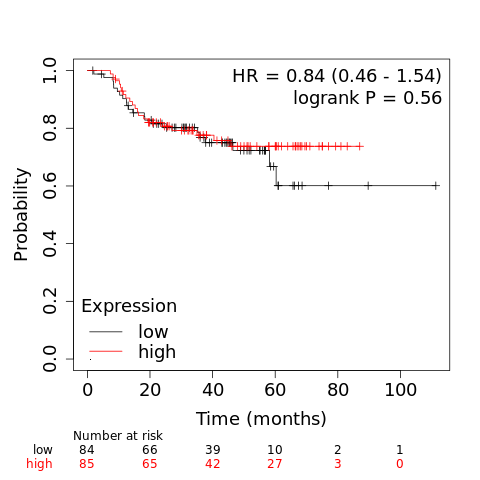
**

Supplementary Figure S11. Kaplan-Meier distant metastasis-free survival plot for high versus low expression level of gene *GRIP2*, restricted to patients with an ER- tumor who received chemotherapy. The plot was generated using KMPlotter data and is based on a total of 169 breast cancer patients (84 with low and 85 with high *GRIP2* expression) with available clinical information.

**
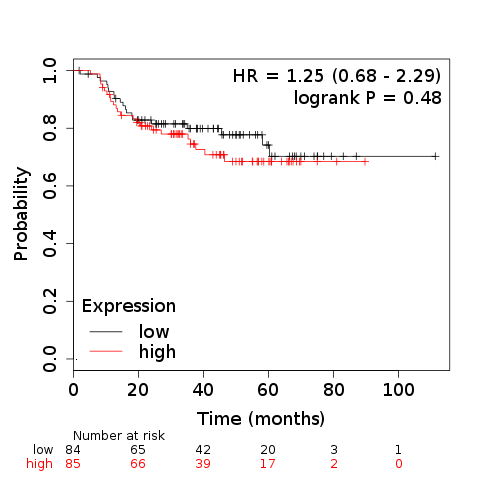
**

Supplementary Figure S12. Kaplan-Meier distant metastasis-free survival plot for high versus low expression level of gene *ARAP2*, restricted to patients with an ER- tumor who received chemotherapy. The plot was generated using KMPlotter data and is based on a total of 169 breast cancer patients (84 with low and 85 with high *ARAP2* expression) with available clinical information.
